# A bibliometric insight into nanomaterials in vaccine: trends, collaborations, and future avenues

**DOI:** 10.3389/fimmu.2024.1420216

**Published:** 2024-08-12

**Authors:** Beibei Wu, Ye Liu, Xuexue Zhang, Ding Luo, Xuejie Wang, Chen Qiao, Jian Liu

**Affiliations:** ^1^ Department of Information, Xiyuan Hospital, China Academy of Chinese Medical Sciences, Beijing, China; ^2^ Traditional Chinese Medicine (TCM) Big Data Innovation Lab of Beijing Office of Academic Research, Xiyuan Hospital, China Academy of Chinese Medical Sciences, Beijing, China; ^3^ Graduate School, China Academy of Chinese Medical Sciences, Beijing, China; ^4^ Graduate School, Beijing University of Chinese Medicine, Beijing, China

**Keywords:** nanoparticles, vaccine, bibliometric, lipid nanoparticles, cancer, COVID-19

## Abstract

**Background:**

The emergence of nanotechnology has injected new vigor into vaccine research. Nanovaccine research has witnessed exponential growth in recent years; yet, a comprehensive analysis of related publications has been notably absent.

**Objective:**

This study utilizes bibliometric methodologies to reveal the evolution of themes and the distribution of nanovaccine research.

**Methods:**

Using tools such as VOSviewer, CiteSpace, Scimago Graphica, Pajek, R-bibliometrix, and R packages for the bibliometric analysis and visualization of literature retrieved from the Web of Science database.

**Results:**

Nanovaccine research commenced in 1981. The publication volume exponentially increased, notably in 2021. Leading contributors include the United States, the Chinese Academy of Sciences, the “*Vaccine*”, and researcher Zhao Kai. Other significant contributors comprise China, the University of California, San Diego, Veronique Preat, the *Journal of Controlled Release*, and the National Natural Science Foundation of China. The USA functions as a central hub for international cooperation. Financial support plays a pivotal role in driving research advancements. Key themes in highly cited articles include vaccine carrier design, cancer vaccines, nanomaterial properties, and COVID-19 vaccines. Among 7402 keywords, the principal nanocarriers include Chitosan, virus-like particles, gold nanoparticles, PLGA, and lipid nanoparticles. Nanovaccine is primarily intended to address diseases including SARS-CoV-2, cancer, influenza, and HIV. Clustering analysis of co-citation networks identifies 9 primary clusters, vividly illustrating the evolution of research themes over different periods. Co-citation bursts indicate that cancer vaccines, COVID-19 vaccines, and mRNA vaccines are pivotal areas of focus for current and future research in nanovaccines. “candidate vaccines,” “protein nanoparticle,” “cationic lipids,” “ionizable lipids,” “machine learning,” “long-term storage,” “personalized cancer vaccines,” “neoantigens,” “outer membrane vesicles,” “*in situ* nanovaccine,” and “biomimetic nanotechnologies” stand out as research interest.

**Conclusions:**

This analysis emphasizes the increasing scholarly interest in nanovaccine research and highlights pivotal recent research themes such as cancer and COVID-19 vaccines, with lipid nanoparticle-mRNA vaccines leading novel research directions.

## 1 Introduction

Over the past decades, vaccine technology has rapidly advanced, from attenuated live vaccines to inactivated vaccines, subunit vaccines, and most recently, nucleic acid vaccines, each technological advancement has equipped humanity with increasingly robust immunological tools (1), reducing the incidence and mortality rates of various diseases (2). However, the safety of vaccines is accompanied by reduced immunogenicity, necessitating formulations to enhance antigen efficacy (3). Against this backdrop, the emergence of nanotechnology has injected new vigor into vaccine research. Nanomaterials, due to their unique physicochemical properties—such as high surface area, tunable size and shape (4), and potential for surface functionalization—exhibit immense potential in vaccine delivery and immunogenic enhancement (5). They facilitate the phagocytosis and rapid processing by antigen-presenting cells and act as immunostimulatory adjuvants, thereby improving vaccine stability and efficacy. Moreover, they enable targeted immunostimulation, reducing side effects and enhancing the specificity and durability of the immune response (6). Certain studies on nanovaccines have achieved remarkable success (7–9). For instance, mRNA nanovaccines have emerged as crucial tools in addressing the global COVID-19 pandemic, significantly contributing to the success of COVID-19 vaccines (7, 8).

Additionally, as our understanding of the immune system deepens, there is growing interest in therapeutic vaccines. Cancer vaccines, known for eliciting persistent immune memory and specific responses to antigens, garner considerable attention (10). Nevertheless, traditional cancer vaccines face limitations in antigen delivery and immune stimulation, often failing to induce a sufficiently robust immune response (11). Nanotechnology offers new perspectives and methods for developing cancer vaccines. It enhances antigen stability (12) and the efficiency of tumor antigen presentation (13). Additionally, nanoparticles can be engineered to target antigens to specific immune cells or the tumor microenvironment, thereby augmenting the immune system’s recognition and response to tumor antigens (14).

The importance of nanovaccines extends beyond improving traditional vaccines to their potential applications in preventing emerging infectious diseases. Traditional vaccines often face challenges such as limited efficacy and long production cycles when dealing with rapidly mutating viruses like influenza and coronaviruses. In contrast, nanovaccines, with their flexible design and rapid production capabilities, can more effectively address these challenges, providing broader and more durable protection (15, 16). Additionally, nanovaccines can be administered through various routes (for example, oral, and nasal spray), enhancing patient acceptance and compliance (17, 18). In conclusion, the application of nanomaterials in vaccine development is propelling vaccine science into a new era. Through continuous exploration and optimization, nanovaccines are poised to play a pivotal role in preventing infectious diseases, treating cancer, and preventing chronic diseases, offering robust protection for human health.

The Bibliometric analysis employs statistical methods to elucidate the results and impact of scholarly research (19), facilitating scholars’ understanding of a discipline’s background, historical processes, fundamental knowledge, current status, developmental directions, and prospects, while also assessing collaborative networks among researchers. It holds significant importance in formulating research policies, guiding journal selection, and facilitating academic evaluations (19–22). Visual knowledge graphs precisely identify pivotal articles across numerous publications, systematically organizing existing research within a field. This approach offers a valuable, timely, replicable, and adaptable methodology (23).

The literature volume in the nanovaccine domain is rapidly expanding, necessitating alignment with emerging trends and critical shifts in collective knowledge development. Therefore, this study utilizes bibliometric analysis to delineate the historical trajectory of nanovaccine research, unveil global collaborative networks, and identify research hotspots. This approach permits a more quantifiable, objective, and comprehensive analysis of the field. To our knowledge, this represents the first bibliometric analysis conducted on nanovaccine research to address the ensuing inquiries:

Q1 - What is the overarching trajectory of research concerning nanovaccine?

Q2 - Which nations, institutions, journals, authors, and funders exert preeminent influence within this research purview?

Q3 - What are the primary disease areas of application for nanovaccine research, and what types of nanomaterials are utilized?

Q4 - What are the pivotal research themes within this domain? Which studies are regarded as seminal milestones? What are the current research hotspots? What are the potential future research directions?

## 2 Materials and methods

### 2.1 Data sources and collection

This study utilized the Web of Science(WOS) as its primary database due to its coverage of over 12,000 academic journals, housing more than 74.8 million scholarly records across 254 disciplinary fields, with 1.5 billion references since 1900 (24). It encompasses a significant corpus of high-quality academic literature, providing robust data support, and is widely used by researchers. Additionally, compared to databases such as Scopus, Medline, and PubMed, WOS is well-suited for various bibliometric analysis tools (25) (for example, citespace) and serves as a principal data source for bibliometric analysis.

To mitigate potential errors from database updates, we promptly acquired all source data from the WOS Core Collection within a single day (November 4, 2023). Given concerns about redundancy and peripheral literature with “topic” retrieval, and after reviewing relevant literature (26) and the MeSH database, our study opted for “title” retrieval, unanimously agreed upon by all authors. The search strategy used was: (TI=(“vaccine*” or “vaccin*”)) AND TI=(nano*). Our data collection period spanned from January 01, 1965, to November 4, 2023, focusing solely on “article” and “review” document types. Other types of documents, such as meetings, book, editorial material, non-peer-reviewed materials, and similar, have been excluded as per database definitions. Language restrictions were not applied. The file format was confined to “Plain text”, and record content was specified as “Full Record and Cited References”. Additionally, to enable comparative analysis across developmental stages and different countries in the nanovaccine field, we exported literature datasets categorized by year, and the top five countries and institutions by publication volume, all on the same day. Funding data for the nanovaccine domain was also exported via the WoSCC online analysis platform concurrently.

### 2.2 Bibliometric analysis and visualization

Microsoft Excel 2019: Used to generate line graphs, bar graphs, and tables for identifying pivotal countries/regions, institutions, authors, journals, funding sources, literature, references, and keywords. The R² test predicted the relationship between the publication year and output.

CiteSpace (https://citespace.podia.com/download, R6.1.6): CiteSpace is designed to identify emerging trends and abrupt changes in scientific literature. Employed for burst analysis and clustering of keywords and references, and network layer overlay of journals.

VOSviewer (https://www.vosviewer.com/, R1.6.18): Used to visually represent co-occurrence networks of countries, institutions, journals, authors, and keywords, as well as co-citation networks of authors and journals. It also facilitates an overlay network to highlight prevailing research themes across significant periods and among key countries and institutions. Simultaneously, it constructs a co-occurrence network of journal-keyword associations, thereby elucidating the primary research subjects of notable journals.

R-bibliometrix (version R 4.3.0): Utilized R-bibliometrix and R-studio to obtain detailed publication trends for countries, institutions, authors, and journals. Moreover, metrics such as the H-index, G-index, and M-index were computed for countries, journals, and authors, alongside the generation of thematic maps.

Scimago Graphica (Setup 1.0.35): An essential tool for generating global maps and institutional networks, primarily used in this study for the geographical visualization of national and institutional collaboration networks. It showcased the overall publications from various countries and institutions.

Pajek (http://mrvar.fdv.uni-lj.si/pajek/) provides various layout algorithms and beautification options for VOSviewer, enhancing the clarity and presentation of network structures. In this study, it was mainly used for the layout optimization of keyword co-occurrence networks.

Additional details are available in **Supplementary Material 1**.

## 3 Results

### 3.1 Global publishing trend

**Figure 1:**
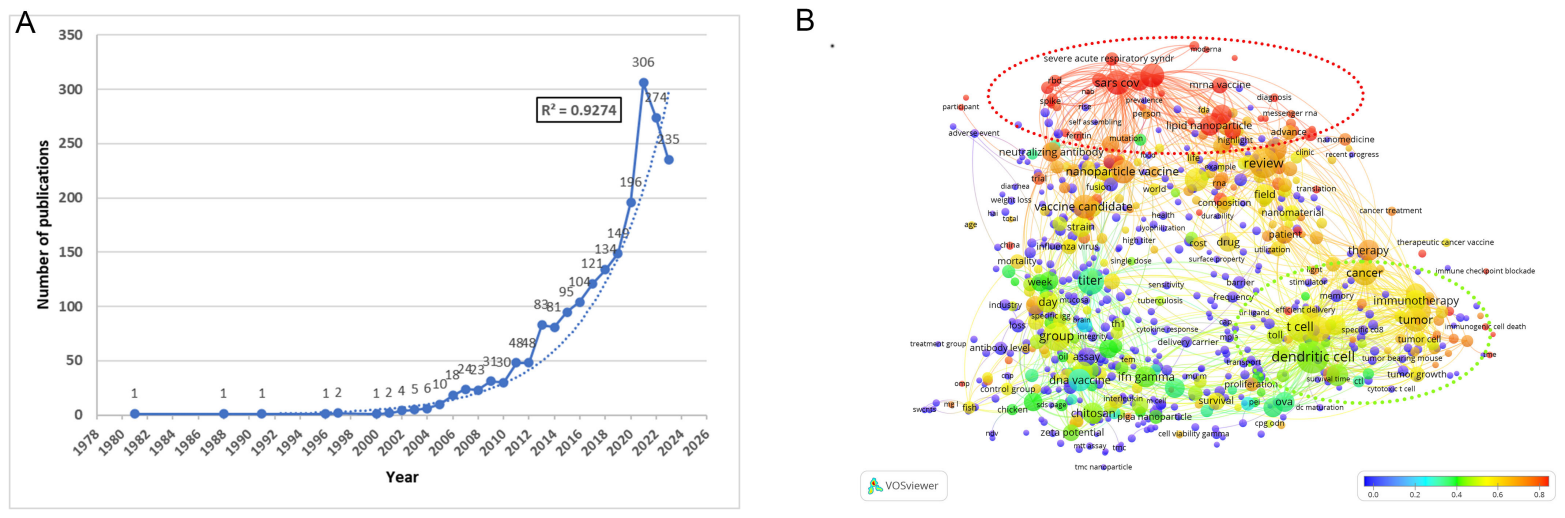
Annual trends and topics of global publications. **(A)** Annual publications and the fitting equation for annual publications. The trend of annual publications is fitted using the exponential trendline type in Excel, where a higher R² value indicates a more precise alignment between the regression line and observed values. **(B)** Term contributions in nanovaccine research from 2020 to 2023. The terms were extracted from the title and abstract. Each node symbolizes a term, with the node’s dimensions mirroring its overall frequency. The color designates the frequency of each term in the period from 2020 to 2023 relative to its overall frequency. The proportion associated with each color is depicted at the bottom right of the figure. The closer the color is to red, the higher the proportion. For example, “lipid nanoparticle” appeared 128 times from 1981 to 2023, with 111 occurrences between 2020 and 2023, constituting 86.72%, and is therefore shown in red on the graph. The interpretation of this graph can be enhanced by considering both the size and color of the nodes: the larger and redder the node, the more prominent the term was as a research focus from 2020 to 2023. The detailed methodology is provided in **Supplementary Material 1**..

The annual publication output provides valuable insights into developmental trends within the research field. Using the stated approach, we retrieved 2034 publications, with an exponential growth trend (R² = 0.9274) in nanovaccine research. We divided the research into four phases, reflecting annual publication trends. From 1981 to 2000, the field saw limited development, with only seven articles published, marking the “preparation period.” The second phase (2000 to 2012) exhibited a gradual upward trend with a moderate growth curve. The third phase (2013 to 2019) indicated an accelerated growth trend, suggesting a stable and positive trajectory. The fourth phase (2020-2023) saw a surge, surpassing 196 annual publications and reaching 1,011 articles (**Figure 1A**), indicating that nanovaccine research has entered a rapid development stage. The term overlap illustrates that post-2020 research predominantly focuses on terms such as Severe Acute Respiratory Syndrome Coronavirus 2(SARS-CoV-2), mRNA vaccine, and LNPs, with their contribution proportions exceeding 80%. Simultaneously, contributions in terms of cancer vaccine and cancer immunotherapy also surpass 50%, highlighting these as focal points of nanovaccine research in the past three years (**Supplementary Material 2**; **Figure 1B**).

### 3.2 Global contribution in nanovaccine research

#### 3.2.1 Co-countries analysis

**Figure 2:**
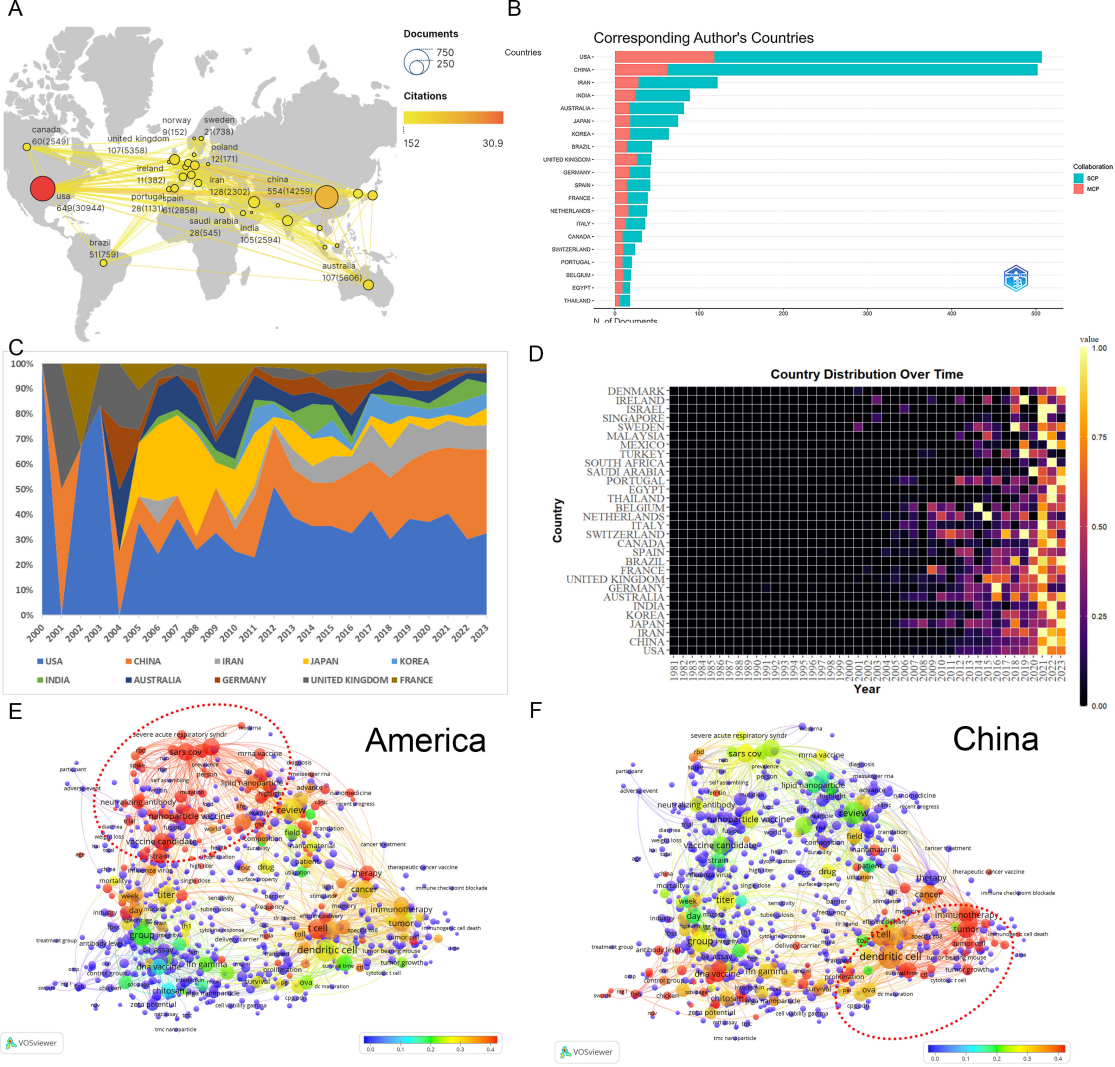
Analysis of countries. **(A)** A world map displaying the total publications from the top 30 countries/regions. The circle size represents the volume of publications, while the circle color indicates the volume of citations. The line thickness denotes the strength of collaboration, highlighting particularly robust efforts between China and the USA. **(B)** Number of documents from corresponding authors’ countries. SCP, Single Country Publications; MCP, Multiple Country Publications. **(C)** Annual proportion trends for the top 10 countries’ publications. The color of the strip represents different countries, and the width of the strip represents their proportion of publication. **(D)** Yearly occurrences of the top 30 countries. The colors represent the publication volume, denoting the strength of the research activity. Brighter colors signify higher publication volume. **(E)** Term contributions of the USA in nanovaccine research. Each node represents a term, with the color denoting the frequency in articles from the USA relative to its overall frequency. The closer the color is to red, the higher the proportion. The larger and redder the node, the more prominent the role of the USA in global research pertaining to that term. **(F)** Term contributions of China in nanovaccine research. Each node represents a term, with the color denoting the frequency in articles from China relative to its overall frequency. The closer the color is to red, the higher the proportion.

Analysis of co-authorship involving countries, institutions, and authors facilitates the identification of potential partners, thereby providing valuable insights for acquiring academic resources, fostering scholarly collaborations, and evaluating academic outcomes. A total of 85 nations actively contribute to nanovaccine research. Key contributors include the USA (649) and China (554) (**Figure 2A**). Publications from other countries or regions numbered fewer than 130, including Iran (128), Australia (107), and India (105). The USA dominates with the highest citations (30,944), total link strength (TLS, 382), h-index (144), and m-index (6). International collaboration shows relatively fewer joint publications (26.11%)(**Figure 2B**), with USA-centric collaborations occupying nine of the top ten partnership positions, mainly with China (**Supplementary Material 3**). Analysis of annual publication volumes and thematic trends across different nations elucidates the enduring dominance of the USA, particularly in fields such as COVID, LNPs, and mRNA vaccines. China demonstrated steady growth in the field from 2000 to 2010, emerging into a pivotal role beyond 2011. Its scholarly focus gravitates towards domains encompassing tumor immunotherapy, DNA vaccines, dendritic cells, and cytotoxic T lymphocytes (**Figures 2C–F**) (**Supplementary Material 2**).

#### 3.2.2 Co-institutions analysis

**Figure 3:**
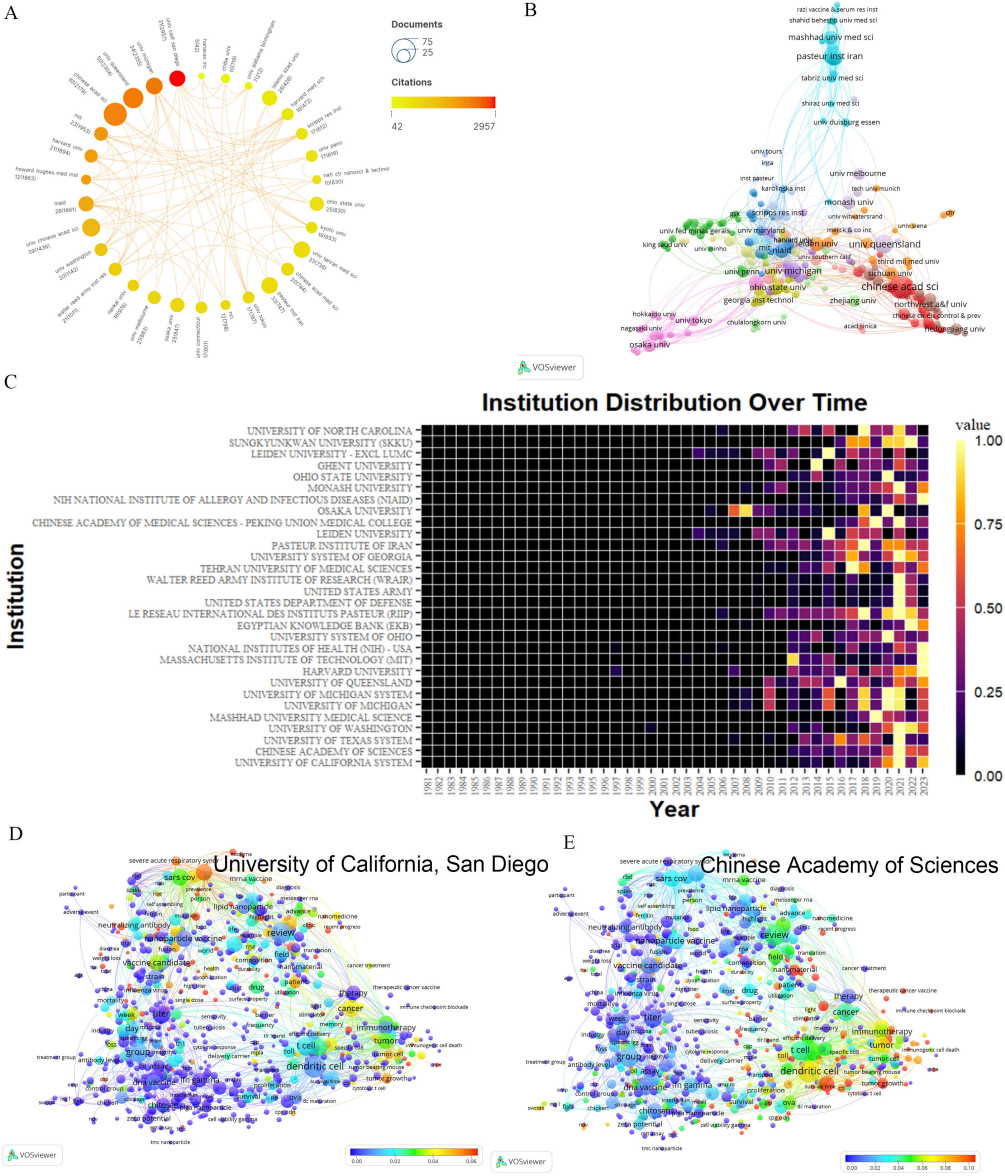
Analysis of institutions. **(A)** A network displaying the total publications from the top 30 institutions. The circle size represents the volume of publications, while the circle color indicates the volume of citations. The interpretation of this graph can be enhanced by considering both the size and color of the nodes: the larger and redder the node, the greater the impact of this institution in the field of nanovaccines. **(B)** Map of co-occurring institutions. Each node represents an institution, node size corresponds to the number of articles, link thickness represents the strength of collaboration, and colors indicate different collaboration groups. For instance, Mashhad University of Medical Sciences and Pasteur Institute Iran are colored blue, signaling their intensive collaboration in nanovaccine research. **(C)** Yearly occurrences of the top 30 institutions. The colors represent the publication volume, denoting the strength of the research activity. Brighter colors signify higher publication volume. **(D)** Term contributions of the University of California, San Diego in nanovaccine research. Each node represents a term, with the color denoting the frequency in articles from the University of California, San Diego relative to its overall frequency. The closer the color is to red, the higher the proportion. **(E)** Term contributions of Chinese Academy of Sciences in nanovaccine research. Each node represents a term, with the color denoting the frequency in articles from the Chinese Academy of Sciences relative to its overall frequency. The closer the color is to red, the higher the proportion.

The top ten contributors are predominantly from China and the USA. The Chinese Academy of Sciences leads with 65 articles and the highest TLS(69). Despite ranking seventh in output, the University of California, San Diego, excels in citation frequency (2957) (**Supplementary Material 4**). Howard Hughes Medical Institute, though producing fewer papers, stands out for its high citation rates. Conversely, the Pasteur Institute of Iran and the Tehran University of Medical Sciences exhibit high outputs but lower citations (**Figure 3A**). Geographically proximate institutions demonstrate greater collaboration ease (**Figure 3B**). Examples include the collaboration between the Chinese Academy of Sciences and Heilongjiang University, the University of Tokyo, and Osaka University, and the collaboration between the Massachusetts Institute of Technology and Harvard University. An in-depth analysis of the Chinese Academy of Sciences’ collaboration network reveals predominant partnerships with institutions specializing in engineering and chemistry. Notably, there is a collaboration with the National Center for Nanoscience and Technology, initially focusing on HIV vaccine development and more recently on cancer nanovaccines and COVID-19 vaccines. Significant collaborations with medical institutions are also evident, such as early-stage research with the Chinese Center for Disease Control and Prevention in HIV nanovaccine development, and recent work with Fujian Medical University on cancer nanovaccines. Moreover, a separate comprehensive analysis of the University of California, San Diego’s collaboration network indicates a broader scope of international cooperation, including recent research with Chiba University on nasal nanovaccines. **Figure 3C** reveals a significant increase in publication volume for most institutions in 2021, with high recent output from institutions like Harvard University, Massachusetts Institute of Technology, National Institutes of Health, and the Egyptian Knowledge Bank. Additionally, **Figures 3D, E** show that the Chinese Academy of Sciences (leading in publication volume) focuses more on cancer and dendritic cell-related fields, while the University of California, San Diego (leading in citation rate) emphasizes research in areas like nanovaccines of COVID-19 and cancer. These emphasis align with each country’s overall research focus (**Supplementary Material 2**).

#### 3.2.3 Co-author analysis

**Figure 4:**
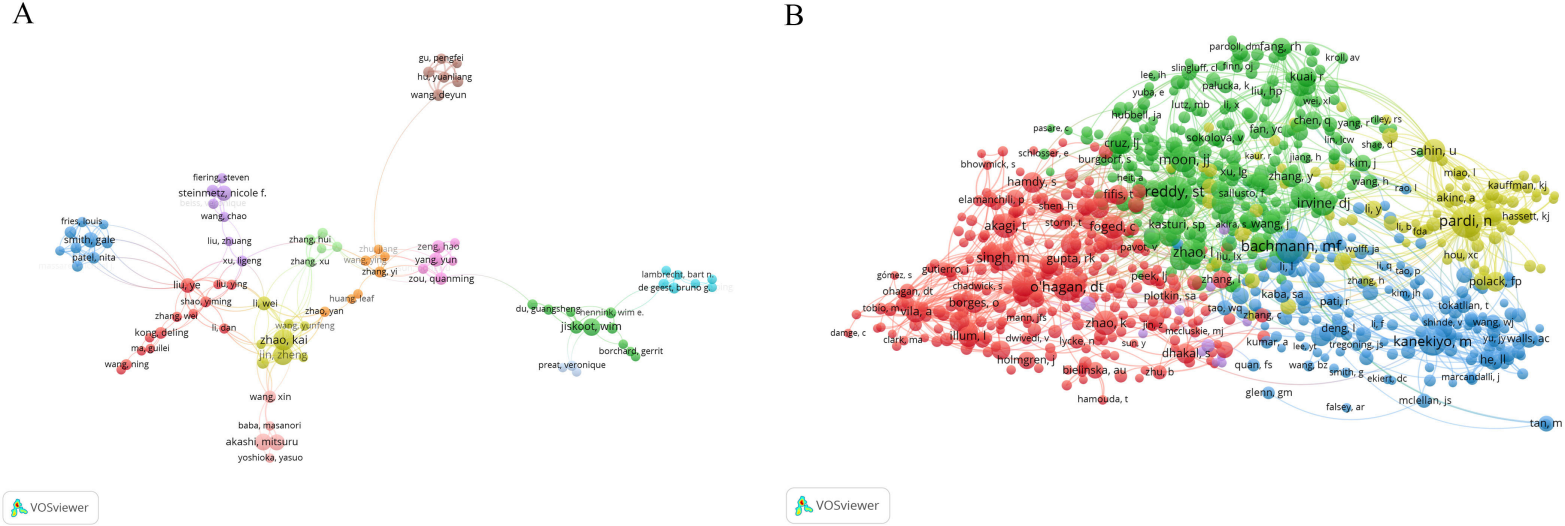
Analysis of authors. **(A)** Map of co-occurring authors. Each node represents an author, node size corresponds to the number of articles, link thickness represents the strength of collaboration, and colors indicate different collaboration groups. **(B)** Map of co-cited authors. Each node represents a co-cited author, node size corresponds to the number of co-citations, links indicate shared co-citations among authors, and colors indicate different clusters of co-cited authors..

This analysis reveals 10,916 contributors, with 249 authors having over five articles. **Figure 4A** illustrates the formation of various collaborative teams in this field. Notable authors include Zhao Kai (25) and Jin Zheng (21) from Heilongjiang University, who constitute a research team primarily dedicated to the study of the Newcastle disease virus and chitosan nanoparticles (**Supplementary Material 5**). Wim Jiskoot (1818) of Leiden University garners the highest citations, with a predominant focus on chitosan nanospheres, Poly(lactic-co-glycolic acid)(PLGA), and nasal vaccination, highlighting his substantial influence and high-caliber research contributions. Additionally, Preat Veronique, Garinot Marie, and Xu Ligeng, despite a publication count of only 5-7 articles, rank among the top four in citation frequency, showcasing their high-quality contributions. Veronique Preat and Marie Garinot collaborate as a research team dedicated to the study of oral vaccines, mrna vaccines and LNPs. Liu Ye has the highest h-index (30), affiliated with the Chinese Academy of Sciences with a predominant focus on the HIV-1 vaccine (**Table 1**).

**Table 1:** The top 10 authors contributed to citations on nanovaccine research.

Rank	Author	Documents	Citations	TLS	H_ Index	M_ Index	Institution	Country
1	jiskoot, wim	17	1818	25	15	0.882	Leiden university	Netherlands
2	preat, veronique	6	1775	6	1	0.056	Katholieke Universiteit Leuven	Belgium
3	garinot, marie	5	1275	2	3	0.167	Sanofi R&D	France
4	xu, ligeng	7	1028	17	5	0.417	Soochow university	China
5	steinmetz, nicole f	13	942	19	12	1.5	University of California, San Diego	USA
6	zhao, kai	25	866	65	16	1.231	Heilongjiang university	China
7	slutter, bram	10	812	19	10	0.667	Leiden university	Netherlands
8	liu, ye	12	799	38	19	1.583	University of Chinese Academy of Sciences	China
9	liu, zhuang	5	726	6	8	0.727	Soochow university	China
10	akashi, mitsuru	16	710	24	14	0.737	Osaka university	Japan

TLS, total link strength. For a given item, the Links and Total link strength attributes indicate, respectively, the number of links of an item with other items and the total strength of the links of an item with other items. In the co-authorship network of researchers, the Total link strength attribute indicates the total strength of the co-authorship links of a given researcher with other researchers.

H-index, an academic’s h-index refers to the maximum number of h for which they have published h papers each cited at least h times. The h-index provides a relatively precise gauge of an individual’s scholarly accomplishments. A higher h-index signifies a greater influence on one’s publications.

M-index, by dividing the h-index by the number of years publishing, accounts for differences in professional longevity.

Co-citation analysis, a methodology in which a third author or paper concurrently cites two authors or papers, elucidates these intricately interwoven associations. A total of 50,015 authors have been co-cited (**Figure 4B**). **Table 2** illustrates the clustering information of co-cited authors analyzed using CiteSpace. By analyzing co-cited author clusters, researchers can discern highly co-cited scholars and their impact within a specific field. For instance, Bachmann MF wields substantial influence in PLGA nanoparticle research, whereas Zhao L commands significant authority in mRNA nanovaccine research.

**Table 2:** Information on the top 7 clusters in the co-cited authors’ network.

Cluster	Size	Silhouette	mean(Year)	Top Term(log-likelihood ratio)	The top 3 co-cited authors
0	228	0.769	2007	chitosan nanoparticle	Ohagan DT(183),Slütter B(100),Neutra MR(77)
1	183	0.762	2012	plga nanoparticle	Bachmann MF(245),Reddy ST(205),Singh M(139)
2	135	0.735	2018	situ cancer vaccination	Wang C(112),Zhang Y(80),Kuai R(78)
3	132	0.836	2020	cov-2 ferritin nanoparticle vaccine	Kanekiyo M(136),Pati R(71),Krammer F(55)
4	128	0.763	2015	mrna vaccine	Zhao L(165),Irvine DJ(141),Liu Y(117)
5	106	0.938	2020	lipid nanoparticle	Pardi N(147),Sahin U(106),Polack FP(94)
6	77	0.93	2006	grass carp	Zhao K(84),Wang Y(54),Cui ZR(43)

“Size” denotes the membership count within a cluster (for example, in co-cited authors analysis, it represents the tally of co-cited authors). “Silhouette” is a metric that assesses the uniformity of the entire cluster; a higher value indicates greater similarity among its members. “mean(Year)” represents the average publication year of members within a cluster, providing insight into the temporal distribution of citations for co-authors within that cluster. The final column lists cluster names derived through the LLR algorithm.

#### 3.2.4 Journals

**Figure 5:**
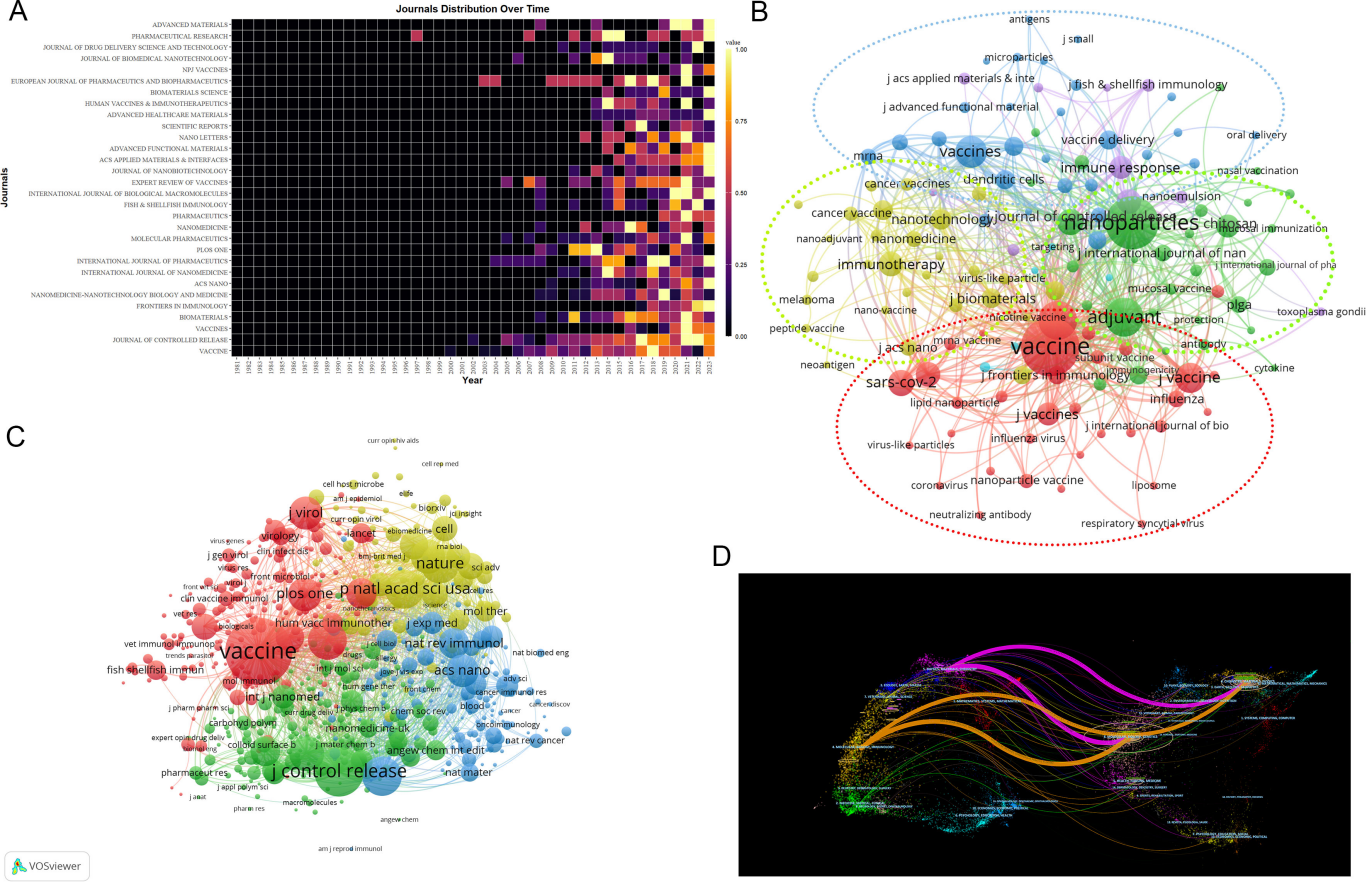
Analysis of journals. **(A)** Yearly occurrences of the top 30 journals. The colors represent the publication volume, denoting the strength of the research activity. Brighter colors signify higher publication volume. **(B)** Co-occurrence network of journal-keyword. Nodes represent either keywords or journals (identified by an uppercase “J” preceding the journal names). Links signify their correlation within the co-occurrence network. Colors indicate different clusters, wherein nodes within each cluster are closely interconnected, facilitating the exploration of thematic domains covered by the journals. For instance, “J Biomaterial” and “virus-like particle” belong to the same cluster, indicating that *Biomaterial* has published numerous articles related to “virus-like particles”. The keywords in this figure consist solely of author keywords. **(C)** Map of co-cited journals. Each node represents a co-cited journal, node size corresponds to the number of co-citations, links indicate shared co-citations among journals, and colors indicate different clusters of co-cited journals. **(D)** The OverlayMaps of journals. On the left is the citing graph, and on the right is the cited graph. The curves represent citation links. In the left graph, the longer the horizontal axis of the ellipse, the more papers published in the journal; the longer the vertical axis, the more authors involved. A knowledge flow analysis revealed the evolutionary relationships between citing and cited journals.

The criteria for assessing a journal’s influence include publication volume, citation frequency, and Impact Factor (IF). The nanovaccine research spans 515 journals, with major contributors being *Vaccine* (105). Among the top ten journals, only three have an Impact Factor over 10: *Journal of Controlled Release* (IF 10.8), *ACS Nano* (IF 17.1), and *Biomaterials* (IF 14). *Journal of Controlled Release* leads in citations (5,195), with the highest h-index (39) (**Supplementary Material 6**). Moreover, the majority of the top productive and cited journals fall within Q1 and Q2 (**Table 3**). **Figure 5A** highlights the early contributors (*Plos One*), persistent contributors (*Journal of Controlled Release*, *Vaccine*), and recent contributors (*Frontiers in Immunology, Vaccines*). **Figure 5B** illustrates the thematic focuses of journals, emphasizing adjuvants, chitosan, immunogenicity, and PLGA in the *Journal of Controlled Release*(green cluster), cancer immunotherapy in *Biomaterials* and *ACS Nano*(yellow cluster), and various topics including influenza, SARS-CoV-2, chitosan nanoparticles, lipid nanoparticles, and mRNA vaccines in *Vaccines* and *Frontiers in Immunology*(red cluster) (**Supplementary Material 6**).

**Table 3:** The top 10 journals contributed to publications on nanovaccine research.

Rank	Journal	Documents	Citations	TLS	H_index	M_index	IF/JCR (2022)	Publisher Country
1	*Vaccine*	105	4624	689	38	1.583	5.5/Q2	Netherlands
2	*Journal of Controlled Release*	81	5195	660	39	1.773	10.8/Q1	Netherlands
3	*Vaccines*	68	1206	475	17	2.125	7.8/Q1	Switzerland
4	*Biomaterials*	52	3340	483	36	2.25	14/Q1	England
5	*Frontiers In Immunology*	45	934	306	13	1.857	7.3/Q1	Switzerland
6	*Nanomedicine-Nanotechnology Biology And Medicine*	45	1282	308	22	1.375	5.4/Q2	Netherlands
7	*Acs Nano*	38	3680	378	24	1.6	17.1/Q1	USA
8	*International Journal of Nanomedicine*	38	1095	220	20	1.429	8/Q2	Netherlands
9	*International Journal of Pharmaceutics*	37	1709	241	19	0.95	5.8/Q1	Netherlands
10	*Plos One*	37	1662	249	22	1.375	3.7/Q2	USA

TLS: In the co-occurrence network of journals, the Total link strength attribute indicates the total strength of the co-authorship links of a given journal with other journals.

IF, impact factor; JCR, Journal Citation Reports.

Co-citation analysis identifies 8,471 co-cited journals, with *Vaccine* (1,557), *Journal of Controlled Release* (4,026), and *Biomaterials* (2,582) being the most frequently cited, all with over 2,500 citations. (**Figure 5C**). OverlayMaps of journals show that those focusing on chemistry, materials, physics; molecular biology, genetics are frequently cited by physics, materials, chemistry; molecular biology, immunology journals, highlighting interdisciplinary connections in nanovaccine research (**Figure 5D**).

#### 3.2.5 Funding agencies

**Figure 6:**
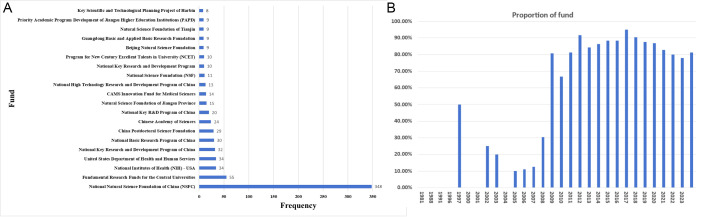
**(A)** The top 20 funding sources in nanovaccine research. **(B)** The annual proportion of articles with funding in nanovaccine research.

Funding support in academic publications is evident, comprising 81.2% (1,651 out of 2,034). The funding sources are diverse, with 200 distinct organizations providing financial support for more than six articles in nanovaccine research, with the majority originating from China. Analysis of WOS funding data reveals that 348 publications received support from the National Natural Science Foundation of China, surpassing other funding sources, and 55 publications received support from Fundamental Research Funds For The Central Universities (**Figure 6A**). Since 2014, over 80% of published articles in the nanovaccine field have received financial support (**Figure 6B**), emphasizing funding’s pivotal role in driving research advancement. Analysis of funding distribution across countries reveals that articles from the USA received support at 85.23%, while those from China received a higher rate of 92.83%, underscoring China’s substantial financial backing for nanovaccine research. Articles from Iran received support at 61.72%, from India at 62.86%, from Australia at 78.13%, and from Japan at 75%, illustrating various national support in nanovaccine research. Analysis of funding distribution across institutions reveals that articles from the Chinese Academy of Sciences received support at 95.39%, while those from the University of California System received a higher rate of 96.14%.

### 3.3 Analysis of keywords

**Figure 7:**
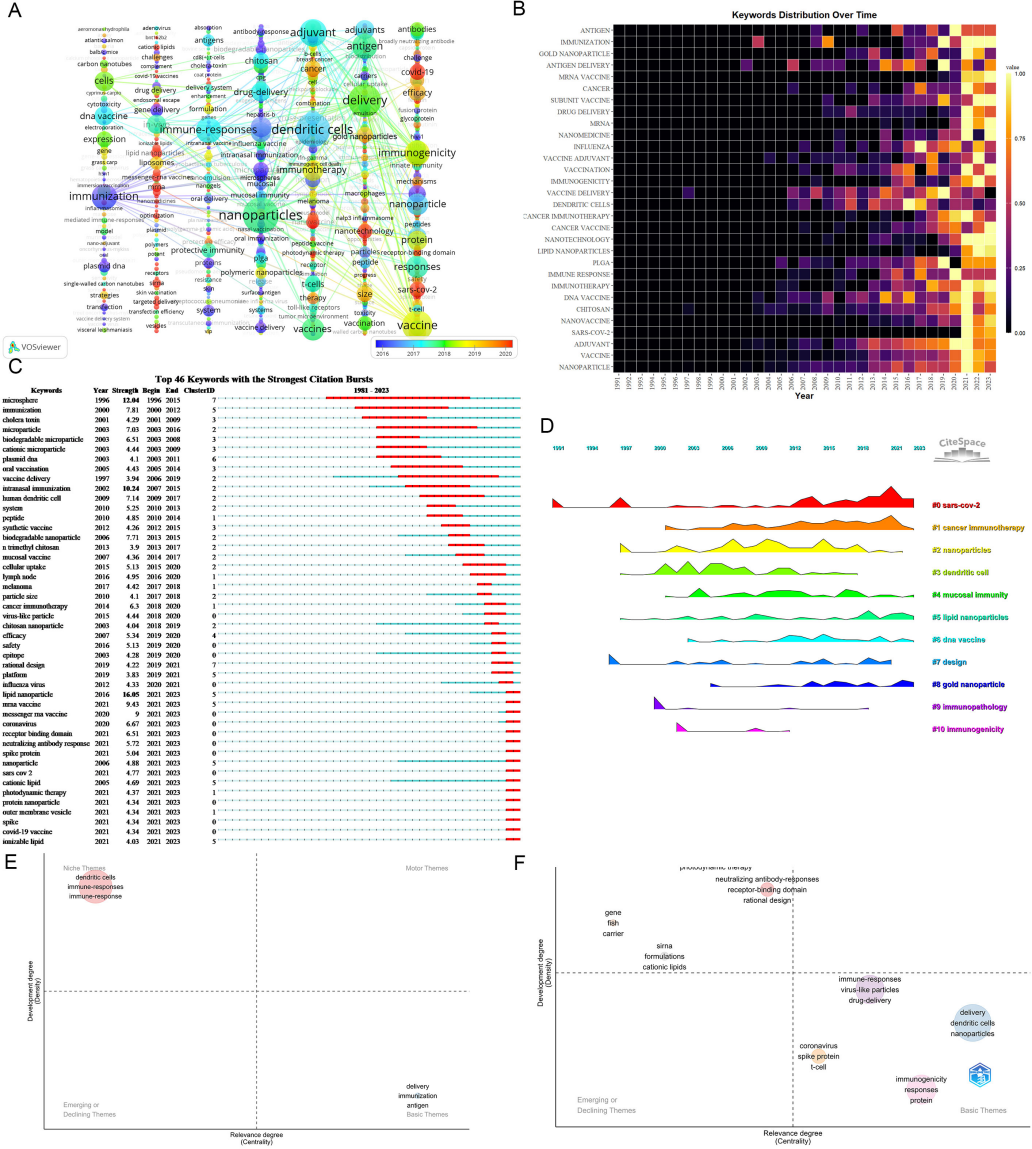
Analysis of keywords. **(A)** Occurrence network of keywords. Nodes represent keywords, with their size reflecting frequency. Lines denote connections between keywords, while each column delineates distinct keyword clusters(These clusters are arranged vertically through Pajek, with closely connected keywords within the same cluster). The analysis should consider node size, node color, and the thematic composition of each cluster. Larger and redder nodes indicate a higher recent frequency of the keyword. Notably, the timeline in the bottom right corner elucidates not the inaugural emergence of keywords but rather their mean occurrence chronology. **(B)** Yearly occurrences of top 30 keywords. The colors represent the frequency of keywords, brighter colors signify higher frequencies. **(C)** Burst analysis of keywords. “Strength” delineates the intensity of keyword bursts, where greater strength signifies heightened frequency of keyword appearances over a defined timeframe. “year” signifies the initial emergence of the keyword. “Begin” and “End” denote the commencement and cessation of the keyword burst, respectively, while the red band delineates the period of the burst. For instance, “lipid nanoparticles” first emerged in 2016, experienced a significant surge starting in 2021, and continued to be prominent until 2023. **(D)** Timeline View of Keywords Cluster. In the timeline view, keywords belonging to the same cluster are aligned horizontally. The initial appearance time of keywords is positioned at the top. This timeline visualization distinctly showcases the number of keywords in each cluster and the temporal span of the cluster. **(E)** Thematic maps on nanovaccine from 1981 to 2019. Color-coded bubbles represent relevant keywords, with size correlating to frequency. The x-axis measures topic relevance, and the y-axis indicates bearing strength or density, reflecting maturity. The upper right cluster (Q1) represents motor themes, which are both important and well-developed. The upper left cluster (Q2) shows highly developed and isolated themes. The lower left quadrant (Q3) reflects emerging or declining themes. The lower right quadrant (Q4) features basic and transversal themes. **(F)** Thematic maps on nanovaccine from 2020 to 2023.

Keywords play a pivotal role in summarizing the themes and content of articles. The extraction of 7,402 keywords unveiled the top three: nanoparticles (452), dendritic cells (414), and delivery(386), emphasizing fundamental elements in nanovaccine research. The principal nanocarriers include Chitosan, virus-like particles, gold nanoparticles, microparticles, liposomes, microspheres, PLGA, and lipid nanoparticles. Nanovaccine is primarily intended to address diseases including SARS-CoV-2, cancer (melanoma, breast cancer), influenza, and HIV. Recent high-frequency keywords predominantly revolve around SARS-CoV-2, cancer vaccines (neoantigen, immunogenic cell death), lipid nanoparticles (ionizable lipid), mRNA vaccine, and long-term storage (**Figure 7A**; **Supplementary Material 7**). **Figure 7B** shows increased interest in LNPs since 2019.

Keyword burst detection identifies terms with markedly increased frequencies, signaling emerging research directions. Burst analysis identifies 46 keywords with robust citation bursts, including lipid nanoparticle (16.05, 2021-2023), microsphere (12.04, 1996-2015), and intranasal immunization (10.24, 2007-2015). Recent keywords boasting significant bursts encompass receptor binding domain (2021-2023), coronavirus (2021-2023), mRNA vaccine (2021-2023), and LNPs (2021-2023). Additional terms like “cationic lipid,” “ionizable lipid,” “protein nanoparticle,” and “outer membrane vesicle” have also witnessed notable bursts (**Figure 7C**), indicating future research directions.

Cluster analysis of keywords enables a comprehensive and precise examination of relationships among keywords. Cluster Analysis generated reveals 11 keywords clusters using CiteSpace: #0 SARS-CoV-2, #1 cancer immunotherapy, #2 nanoparticles, #3 dendritic cells, #4 mucosal immunity, #5 lipid nanoparticles, #6 DNA vaccine, #7 design, #8 gold nanoparticle, #9 immunopathology, and #10 immunogenicity. #0, #1, and #2 emerged as the top 3 clusters. **Figure 7D** illustrates the developmental trajectory of keyword clusters, highlighting ongoing advancement in clusters #0, #1, and #5. These continuous advancements underscore the current dynamism of these research domains in nanovaccine studies.

The thematic map serves as a crucial analytical tool, revealing intricate relationships within and between research topics in the field of nanovaccinology. Given the exponential increase in scholarly output in 2020, we partition the thematic progression into two distinct phases: an initial phase (1981-2019) and an accelerated growth phase (2020-2023). **Figures 7E, F** depict the transition from the initial phase to the rapid development stage. The migration of themes related to “dendritic cells” and “immune responses” from Q2 to Q4 indicates their progressively intimate connections with other topics in the nanovaccine domain, assuming foundational themes. Throughout the period spanning 2020 to 2023, immunogenicity and dendritic cells function as basic themes. Furthermore, themes positioned between Q1 and Q2, such as neutralizing antibody responses, receptor-binding domains, and rational design, suggest their potential to evolve into motor themes in the future.

### 3.4 Seminal literature - landmark research

Highly cited literature often signifies impactful and innovative research in its field. Analyzing these studies provides insights into advancements, trends, and future directions in nanovaccine development (**Supplementary Material 8**). Scrutinizing the countries, institutions, and authors associated with this literature identifies key contributors in the nanovaccine domain, aiding global research understanding and fostering future collaborations. Among the top 100 cited works, there are 39 reviews, 59 experiments, and 2 clinical trials (one related to the melanoma vaccine in 2012 and the other concerning the COVID-19 vaccine in 2020). The USA is the primary contributor (39), followed by China (12). The top institutions in terms of publications are the University of California San Diego (18) and the Massachusetts Institute of Technology (17). *ACS Nano* (10) and the *Journal of Controlled Release* (7) are the journals with the highest publication volume. **Table 4** highlights the top 20 articles, including Sai T. Reddy’s 2007 article in *Nature Biotechnology*, which garnered the highest number of citations(954). This study affirmed that nanoparticles can serve as a vaccine platform by targeting lymph node–residing dendritic cells via interstitial flow, activating these cells through *in situ* complement activation, and eliciting both humoral and cellular immunity in mice (**Table 4**). Other highly cited articles predominantly address vaccine delivery carriers, physicochemical properties of nanoparticles, tumor studies, COVID-19 vaccines, adjuvants, and vaccines for various diseases such as hepatitis B, influenza, and malaria.

**Table 4:** The top 20 publications in number of total citations.

Title	Year	Citation	Author	Institution	Country	Journal
Exploiting lymphatic transport and complement activation in nanoparticle vaccines	2007	986	Reddy, ST	École Polytechnique Fédérale de Lausanne	Switzerland	*Nature Biotechnology*
Nanoparticles as potential oral delivery systems of proteins and vaccines: a mechanistic approach	2006	977	Preat, V	Catholic University of Louvain	Belgium	*Journal of Controlled Release*
Cancer cell membrane-coated nanoparticles for anticancer vaccination and drug delivery	2014	954	Zhang, LF	University of California, San Diego	USA	*Nano Letters*
Phase 1-2 trial of a sars-cov-2 recombinant spike protein nanoparticle vaccine	2020	701	Keech, C	Novavax	USA	*New England Journal of Medicine*
Designer vaccine nanodiscs for personalized cancer immunotherapy	2017	699	Schwendeman, A; Moon, JJ	University of Michigan	USA	*Nature Materials*
Nanoparticle vaccines	2014	638	Middelberg, APJ	University of Queensland	Australia	*Vaccine*
Chitosan and chitosan ethylene oxide propylene oxide block copolymer nanoparticles as novel carriers for proteins and vaccines	1997	584	Calvo, P	Universidad de Santiago de Compostela	Spain	*Pharmaceutical Research*
Synthetic nanoparticles for vaccines and immunotherapy	2015	552	Irvine, DJ	Massachusetts Institute of Technology	USA	*Chemical Reviews*
Self-assembling influenza nanoparticle vaccines elicit broadly neutralizing h1n1 antibodies	2013	551	Nabel, GJ	Sanofi	France	*Nature*
Size-dependent immunogenicity: therapeutic and protective properties of nanovaccines against tumors	2004	515	Plebanski, M	Austin & Repatriation Medical Center	Australia	*Journal of Immunology*
Biodegradable nanoparticles are excellent vehicle for site directed *in-vivo* delivery of drugs and vaccines	2011	464	Singh, DK	Winston-Salem State University	USA	*Journal of Nanobiotechnology*
Gold nanoparticles as a vaccine platform: influence of size and shape on immunological responses *in vitro* and *in vivo*	2013	453	Niikura, K	Hokkaido University	Japan	*ACS Nano*
mRNA-lipid nanoparticle covid-19 vaccines: structure and stability	2021	448	Jiskoot, W	Leiden University	Netherlands	*International Journal of Pharmaceutics*
Lipid nanoparticles-from liposomes to MRNA vaccine delivery, a landscape of research diversity and advancement	2021	445	Zhou, QQ	CAS	China	*Acs Nano*
Cancer cell membrane-coated adjuvant nanoparticles with mannose modification for effective anticancer vaccination	2018	428	Xu, LG; Peng, R; Liu, Z	Soochow University	China	*Acs Nano*
Nanotechnology in vaccine delivery	2008	416	Berkland, C	University of Kansas	USA	*Advanced Drug Delivery Reviews*
Covid-19 vaccine development and a potential nanomaterial path forward	2020	377	Steinmetz, NF	University of California, San Diego	USA	*Nature Nanotechnology*
Nanogel antigenic protein-delivery system for adjuvant-free intranasal vaccines	2010	370	Kiyono, H	University of Tokyo	Japan	*Nature Materials*
Vaccine delivery using nanoparticles	2013	364	Gregory, AE	University of Exeter	England	*Frontiers In Cellular And Infection Microbiology*
Low molecular weight chitosan nanoparticles as new carriers for nasal vaccine delivery in mice	2004	351	Alonso, MJ	Philipps University of Marburg	Germany	*European Journal of Pharmaceutics And Biopharmaceutics*

### 3.5 Analysis of co-cited references

#### 3.5.1 Clusters and timeline of research

**Figure 8:**
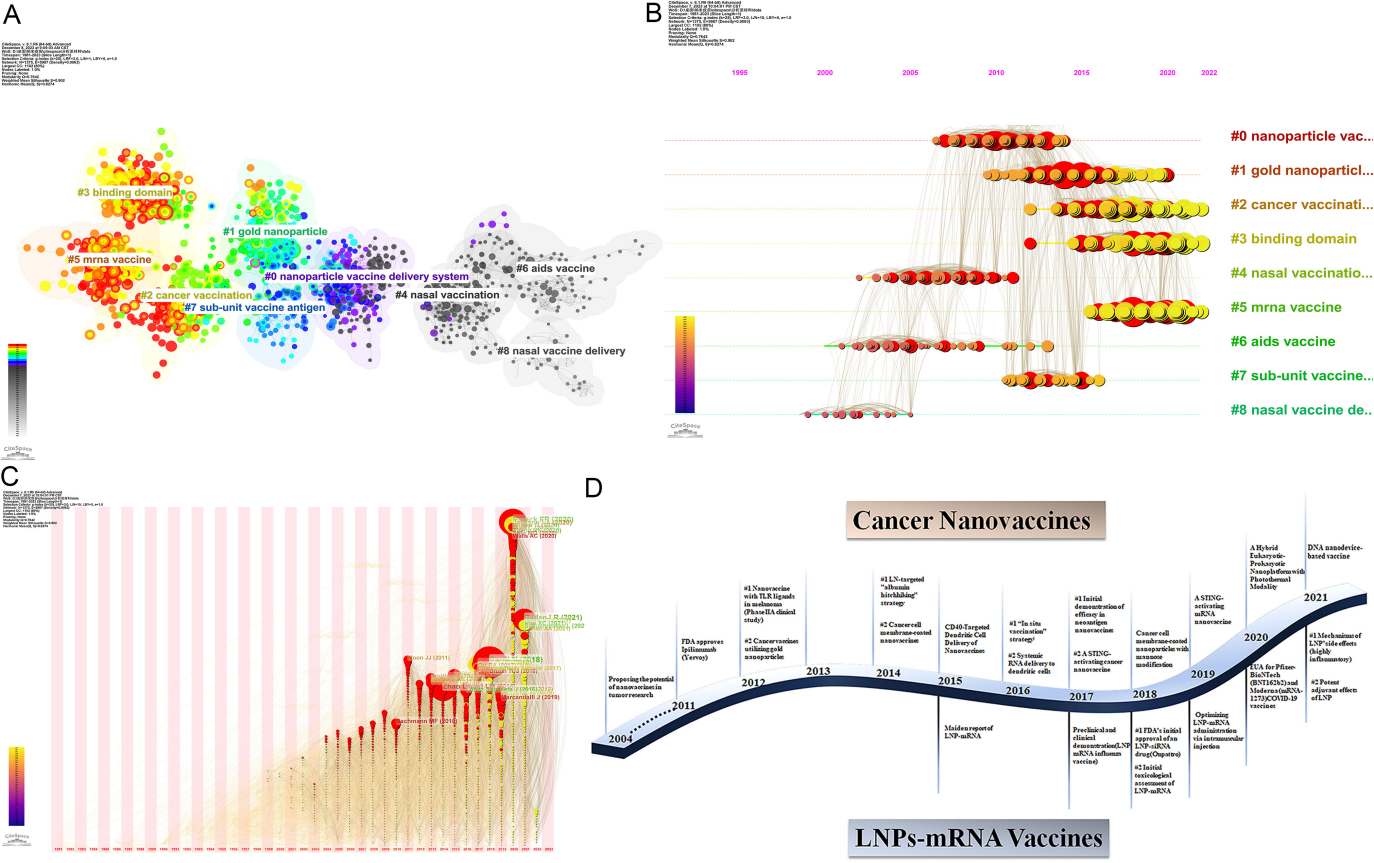
**(A)** Co-cited literature cluster in the nanovaccine domain from 1981 to 2023. The size of network nodes correlates with the frequency of co-citations. Documents within the same cluster exhibit strong connections, whereas those in different clusters are loosely connected. Smaller clusters often lack representativeness compared to larger ones, as they are typically formed by the citation patterns of a few publications. The quality of clusters is further illuminated by their silhouette score, a metric of homogeneity or consistency. Silhouette scores approach 1 for homogeneous clusters; in **Figure 8A**, these clusters are notably homogeneous, each named by title words with the highest log-likelihood ratio (LLR). Each cluster in **Figure 8A** is characterized by a distinct color, signifying a relatively focused appearance over time. The color gradient from left to right in the network transitions from light to dark, symbolizing the progression of research from earlier to more recent phases. The average appearance time of the cluster is indicated in the lower-left corner. **(B)** Timeline View of co-citation Cluster. In the timeline view, references belonging to the same cluster are aligned horizontally. Each horizontal line is labeled with a cluster tag on its far right end. This representation vividly presents the number of references per cluster and the temporal scope of research. A high count of documents within a cluster denotes its significance, while a broad temporal range reflects the duration. Moreover, through the analysis of temporal spans across references, one can scrutinize the phases of emergence, flourishing, and decline in research within the field, thereby fostering discourse on the chronology of scientific inquiry. **(C)** Time zone graph of co-cited literature. The time zone graph consolidates co-cited literature within corresponding periods, reflecting when the literature was initially cited. A significant concentration of aggregated literature citations within a time zone indicates the accumulation of influential outcomes. **(D)** Developmental trends of cancer vaccines and LNPs-mRNA vaccines in the nanovaccine domain(which was generated based on annual burst co-citations and the most highly cited publications).

Co-citation reflects the referencing of two or more papers by other scholarly works, showcasing their interconnectedness within the research domain (27). Through co-citation analysis, it elucidates foundational knowledge and frontiers of academic research, enhancing our understanding of the field’s structure and evolution. CiteSpace was employed for co-citation analysis, resulting in 9 principal clusters. The clustering results (Q = 0.7642, S = 0.902) highlight reliability. **Supplementary Material 9** details these clusters by size and composition. The largest cluster is #0 nanoparticle vaccine delivery system. (**Figure 8A**). The transition from cooler to warmer color gradients visually depicts knowledge flow among these clusters. Additionally, the Timeline of the cluster similarly illustrates research theme evolution across different periods in the co-citation network, highlighting shifts: before 2013, the primary focus was on #4 nasal vaccination, #6 AIDS vaccine, and #8 nasal vaccine delivery; from 2014 to 2017, attention gradually shifted towards #0 nanoparticle vaccine delivery system and #7 sub-unit vaccine antigen; subsequently, from 2017 to 2020, the focus turned to #1 gold nanoparticle and #2 cancer vaccination; finally, from 2020 to the present, emerging fields such as #3 binding domain and #5 mrna vaccine have gradually gained prominence (**Figure 8B**). Moreover, clusters #2, #3, and #5 contain nodes marked with red rings indicating citation bursts. **Table 5** lists key references from these clusters (#2, #3, #5), outlining their critical research focuses. Furthermore, there was a significant surge in citations during 2020, likely linked to the emergence of the COVID-19 pandemic in late 2019 (**Figure 8C**).

**Table 5:** The top 10 co-cited references in #2, #3, and #5 clusters.

Cluster	TI	Freq	Burst	Author	Year	Source
**#2**	Structure-based programming of lymph-node targeting in molecular vaccines	28	13.07 (2015- 2019)	Liu HP	2014	*Nature*
	*In vivo* characterization of the physicochemical properties of polymer-linked TLR agonists that enhance vaccine immunogenicity	24	10.27 (2016- 2020)	Lynn GM	2015	*Nat Biotechnol*
	Systemic RNA delivery to dendritic cells exploits antiviral defence for cancer immunotherapy	45	11.98 (2017- 2021)	Kranz LM	2016	*Nature*
	Designer vaccine nanodiscs for personalized cancer immunotherapy	66	15.25 (2018- 2020)	Kuai R	2017	*Nat Mater*
	An immunogenic personal neoantigen vaccine for patients with melanoma	36	7.17 (2020- 2021)	Ott PA	2017	*Nature*
	A STING-activating nanovaccine for cancer immunotherapy	27	9.72 (2019- 2021)	Luo M	2017	*Nat Nanotechnol*
	Antigen-capturing nanoparticles improve the abscopal effect and cancer immunotherapy	23	8.27 (2019- 2021)	Min YZ	2017	*Nat Nanotechnol*
	Cancer cell membrane-coated adjuvant nanoparticles with mannose modification for effective anticancer vaccination	40	4.42 (2019- 2020)	Yang R	2018	*Acs Nano*
	Towards personalized, tumour-specific, therapeutic vaccines for cancer	32		Hu Z	2018	*Nat Rev Immunol*
	Correlates of adjuvanticity: A review on adjuvants in licensed vaccines	28		Del Giudice G	2018	*Semin Immunol*
**3**	Self-assembling protein nanoparticles in the design of vaccines	37	9.84 (2017- 2021)	López-Sagaseta J	2016	*Comput Struct Biotec*
	Nanoparticle vaccines against infectious diseases	71	9.58 (2020- 2023)	Pati R	2018	*Front Immunol*
	Innate immune recognition of glycans targets HIV nanoparticle immunogens to germinal centers	40	5.37 (2020- 2023)	Tokatlian T	2019	*Science*
	Mosaic nanoparticle display of diverse influenza virus hemagglutinins elicits broad B cell responses	37	4.97 (2020- 2023)	Kanekiyo M	2019	*Nat Immunol*
	Induction of potent neutralizing antibody responses by a designed protein nanoparticle vaccine for respiratory syncytial virus	36	4.88 (2020- 2023)	Marcandalli J	2019	*Cell*
	Cryo-EM structure of the 2019-nCoV spike in the prefusion conformation	46	8.52 (2020- 2021)	Wrapp D	2020	*Science*
	Nanoparticle vaccines based on the receptor binding domain (rbd) and heptad repeat (hr) of sars-cov-2 elicit robust protective immune responses	42		Ma XC	2020	*Immunity*
	Covid-19 vaccine development and a potential nanomaterial path forward	35	8.18 (2021- 2023)	Shin MD	2020	*Nat Nanotechnol*
	Structure, Function, and Antigenicity of the SARS-CoV-2 Spike Glycoprotein	33	8.31 (2020- 2023)	Walls AC	2020	*Cell*
	Rapid development of sars-cov-2 spike protein receptor-binding domain self-assembled nanoparticle vaccine candidates	41	–	Kang YF	2021	*Acs Nano*
**5**	Modified mRNA Vaccines Protect against Zika Virus Infection	29	7.29 (2020- 2023)	Richner JM	2017	*Cell*
	mRNA vaccines — a new era in vaccinology	111	16.47 (2021- 2023)	Pardi N	2018	*Nat Rev Drug Discov*
	Optimization of lipid nanoparticles for intramuscular administration of mrna vaccines	39	8.43 (2021- 2023)	Hassett KJ	2019	*Mol Ther-Nucl Acids*
	The Onpattro story and the clinical translation of nanomedicines containing nucleic acid-based drugs	29	6.26 (2021- 2023)	Akinc A	2019	*Nat Nanotechnol*
	Safety and Efficacy of the BNT162b2 mRNA Covid-19 Vaccine	92	20.03 (2021- 2023)	Polack FP	2020	*New Engl J Med*
	An mRNA Vaccine against SARS-CoV-2 — Preliminary Report	60	–	Jackson LA	2020	*New Engl J Med*
	A Thermostable mRNA Vaccine against COVID-19	39	8.43 (2021- 2023)	Zhang NN	2020	*Cell*
	Efficacy and Safety of the mRNA-1273 SARS-CoV-2 Vaccine	78	16.95 (2021- 2023)	Baden LR	2021	*New Engl J Med*
	Lipid nanoparticles for mRNA delivery	39		Hou XC	2021	*Nat Rev Mater*
	Mrna-lipid nanoparticle covid-19 vaccines: structure and stability	38		Schoenmaker L	2021	*Int J Pharmaceut*

The core components of #2 cancer immunotherapy represent pivotal research associated with cancer vaccines, predominantly featured in esteemed journals such as *Nature* and Nature research journals. In 2014, the Haipeng Liu team (28) introduced the “albumin hitchhiking” strategy, enhancing lymph node enrichment of cancer vaccines through optimized amphiphiles and offering a novel direction for lymph node-targeted vaccines. In 2016, Rui Kuai’s team (13) proposed nano-discs carrying neoantigen peptides and adjuvants, demonstrating potent, broad-spectrum T-cell responses when combined with immune checkpoint inhibitors. This pioneering study validated personalized nanovaccines tailored to tumor neoantigens, laying the foundational groundwork with a citation burst strength of 15.25 from 2018 to 2020. In 2017, Ott, Zhuting Hu’s team reaffirmed the feasibility, safety, and immunogenicity of tumor neoantigen vaccines in cancer patients (29). Recent years have seen burgeoning research and development in STING pathway-based cancer nano vaccines. Jinming Gao’s team in 2017 reported a STING-activating nanovaccine, demonstrating potent anti-tumor efficacy across various cancers (30). Furthermore, in 2014, Ronnie H. Fang’s team introduced a unique therapeutic approach—nanoparticles coated with cancer cell membranes—preserving all antigenic information to address immunogenicity challenges associated with tumors and their antigens, providing an alternative solution for nano-cancer vaccines (31). Subsequent validation has confirmed the efficacy and safety of this method, exemplified by Rong Yang’s team in 2018 with the design of cancer cell membrane-coated adjuvant nanoparticles modified with mannose, eliciting robust anti-tumor immunity (32).

The #5 mRNA vaccine is a recent cluster, with the average publication year of the literature being 2019. Seven out of the top ten articles in the #5 cluster have exhibited citation bursts, all of which have continued until 2023, indicating that it will become an emerging trend in the field. These articles are closely related to SARS-CoV-2 and lipid nanoparticles and are predominantly published in high-impact journals such as *The New England Journal of Medicine*, *Cell*, and various *Nature* sub-journals. Analyzing these research articles within the #5 cluster reveals the developmental trajectory of mRNA nanovaccines and highlights significant key research achievements. The COVID-19 pandemic has significantly advanced clinical trials of nanovaccines for COVID-19, with a notable focus on lipid nanoparticles in mRNA vaccine delivery. However, the success of lipid nanoparticles in mRNA delivery was not achieved overnight. LNPs were first reported as an mRNA delivery system in 2015 (33) and were recognized in 2016 as “the most promising non-viral gene delivery system, critical to the success and clinical translation of mRNA vaccines.” (34). However, this article experienced a citation burst of 9.88 between 2020 and 2021, four years after its publication. In 2017, Kapil Bahl’s team demonstrated the protective immunogenicity of lipid nanoparticle-mRNA vaccines against the influenza virus in both preclinical and clinical settings (35); the same year, similar positive results were obtained in animal experiments targeting Zika virus infection (36). In 2018, an article titled “mRNA vaccines — a new era in vaccinology” was published, indicating that mRNA vaccines are a promising alternative to traditional vaccine approaches (1). This article experienced a citation burst strength of 16.47 from 2021 to 2023. In the same year, the first FDA-approved lipid nanoparticle-siRNA therapeutic, Onpattro (Patisiran), was approved, providing a robust foundation for developing numerous nucleic acid-based nanoparticle delivery therapies (37). Subsequently, Kerry E. Benenato’s team conducted toxicological evaluations of LNPs-mRNA in rats and non-human primates, representing the first evidence of safely repeatable administration at therapeutically relevant levels (38). Additionally, Kimberly J. Hassett proposed the rational evolution and selection of improved formulations of LNP-mRNA for intramuscular administration (39). The above-mentioned research lays a strong foundation for the rapid preparation of LNP-based mRNA nanovaccines during subsequent pandemics.

3 is also the most recently formed cluster, with its core literature marking significant milestones in the development of COVID-19 nanovaccines. In February 2020, the China Novel Coronavirus Investigating and Research Team reported the discovery of the novel coronavirus in *The New England Journal of Medicine*, initiating global research and response to the virus (40). In March, scientists elucidated the structure and antigenic properties of the SARS-CoV-2 spike protein, providing critical information for vaccine design (41, 42). By April, Markus Hoffmann and colleagues had further clarified the mechanism of viral entry into cells, highlighting the pivotal roles of ACE2 and TMPRSS2 in viral invasion (43). Building on these research, COVID-19 nanovaccines entered early development stages. In June, researchers first reported the safety and immunogenicity of a recombinant adenovirus-vectored COVID-19 vaccine in human trials (44). In September, *The New England Journal of Medicine* published findings that the SARS-CoV-2 Recombinant Spike Protein Nanovaccines (NVX-CoV2373), developed by Novavax, was safe and elicited a robust immune response in Phase I/II clinical trials (45). By October, trials funded by BioNTech and Pfizer supported the selection of BNT162b2 for Phase II/III safety and efficacy evaluations (46). By the end of 2020, groundbreaking progress had been made with COVID-19 vaccines, as the mRNA vaccines from BioNTech/Pfizer and Moderna demonstrated over 90% efficacy in Phase III clinical trials, subsequently receiving emergency use authorizations (7, 8).

Through a concise analysis of annually surging citations and the most cited publications, **Figure 8D** illustrates the developmental trends of cancer vaccines and lipid nanoparticle-mRNA(LNP-mRNA) vaccines in the nanovaccine domain.

#### 3.5.2 Most co-cited papers

In the top 10 co-cited publications, articles of cluster #5 mrna vaccine dominate, with other clusters such as #1 gold nanoparticle, #2 cancer vaccination, and #3 binding domain (**Table 6**). The foremost co-cited work is a review authored by Pardi N, published in 2018 in *Nature Reviews Drug Discovery.* This review provides a comprehensive elucidation of basic mRNA vaccine pharmacology and recent advancements in mRNA vaccine technology, particularly in the realms of infectious diseases and cancer, along with an exploration of therapeutic considerations and challenges. This seminal work has garnered 111 co-citations and experienced a citation burst strength of 16.47 between 2021 and 2023. Polack FP’s (7) work, garnering 92 co-citations, details the pivotal clinical trials of BNT162b2, offering reliable data and methodologies that have significantly contributed to COVID-19 nanovaccine development. This study witnessed a citation burst strength of 20.03 between 2021 and 2023. Both belong to cluster #5 mRNA vaccine.

**Table 6:** Top ten co-cited references in nanovaccine research.

Title	Author	Source	Year	Co-citations	Burst	Cluster
mRNA vaccines — a new era in vaccinology	Pardi N	*Nature Reviews Drug Discovery*	2018	111	16.47 (2021-2023)	5
Safety and Efficacy of the BNT162b2 mRNA Covid-19 Vaccine	Polack FP	*New England Journal of Medicine*	2020	92	20.03 (2021-2023)	5
Nanoparticle vaccines	Zhao L	*Vaccine*	2014	86	37.68 (2015-2019)	1
Efficacy and Safety of the mRNA-1273 SARS-CoV-2 Vaccine	Baden LR	*New England Journal of Medicine*	2021	78	16.95 (2021-2023)	5
Nanoparticle Vaccines Against Infectious Diseases	Pati R	*Frontiers in Immunology*	2018	71	9.58 (2020-2023)	3
Designer vaccine nanodiscs for personalized cancer immunotherapy	Kuai R	*Nature Materials*	2017	66	15.25 (2018-2020)	2
An mRNA Vaccine against SARS-CoV-2 — Preliminary Report	Jackson LA	*New England Journal of Medicine*	2020	60	0	5
Cryo-EM structure of the 2019-nCoV spike in the prefusion conformation	Wrapp D	*Chemical Reviews*	2020	46	8.52 (2020-2021)	3
Synthetic Nanoparticles for Vaccines and Immunotherapy	Irvine DJ	*Science*	2015	46	19.75 (2016-2020)	1
Systemic RNA delivery to dendritic cells exploits antiviral defence for cancer immunotherapy	Kranz LM	*Nature*	2016	45	11.98 (2017-2021)	2

#### 3.5.3 Burst analysis

**Figure 9:**
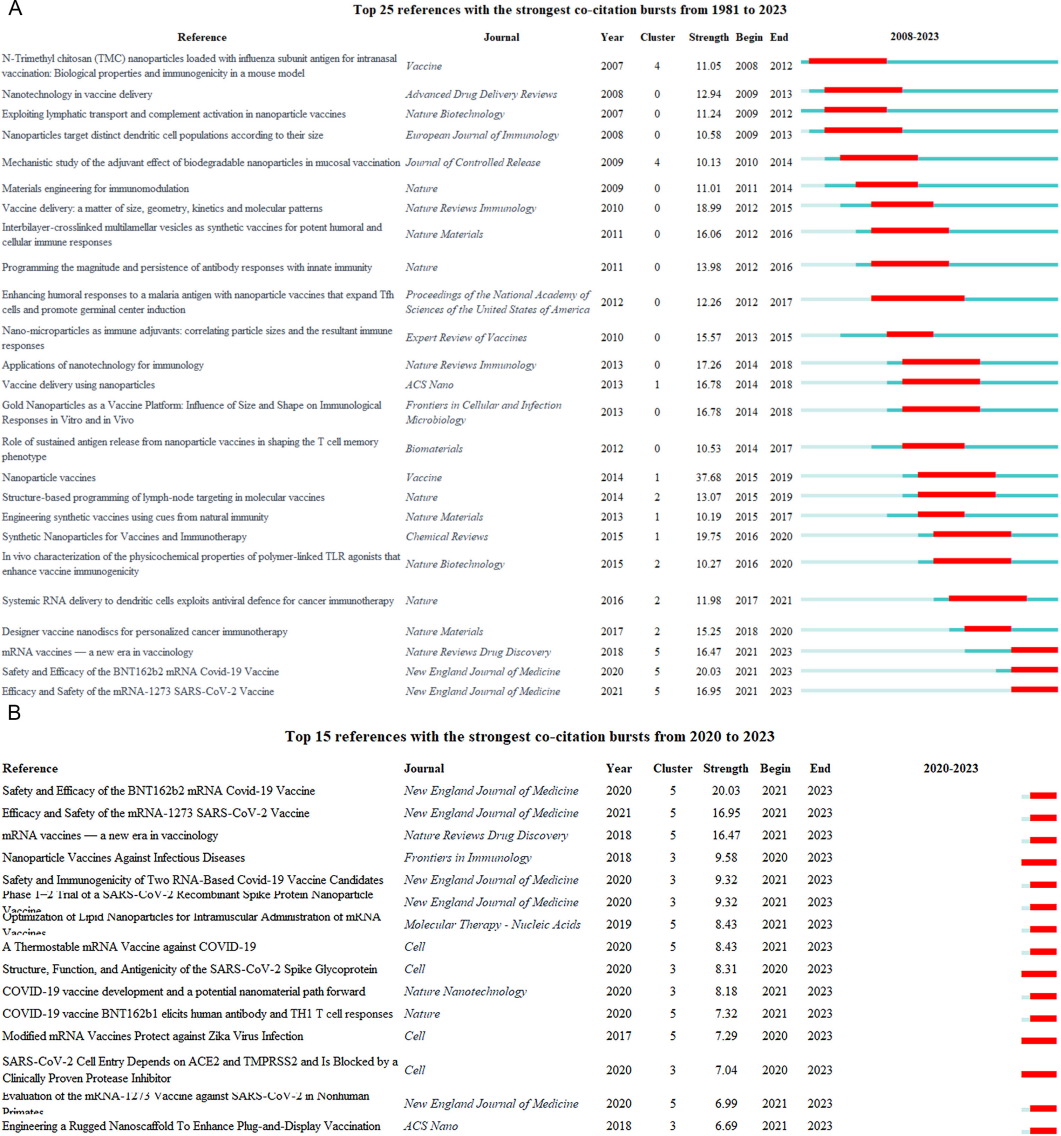
Burst analysis of reference. **(A)** Top 25 references with the strongest co-citation bursts from 1981 to 2023. The blue line represents the timeline spanning from 2008 to 2023, while the red line marks the specific time when each citation burst occurred. **(B)** Top 15 references with the strongest co-citation bursts from 2020 to 2023. The blue line represents the timeline spanning from 2020 to 2023, while the red line marks the specific time when each citation burst occurred.

The eruption of citations has provided a valuable means for observing the evolution of research focal points. A total of 259 references underwent citation bursts(**Supplementary Material 10**). **Figure 9A** displays the 25 articles with the highest citation burst strength, similarly underscoring the flux of comparable thematic trends. The article with the highest citation burst strength was a 2014 review in the *Vaccine*, which delves into prophylactic nanovaccines, encompassing various types of nanoparticles and their interactions with immune cells and biological systems. In CiteSpace, a greater number of burst nodes within a cluster reflects higher field activity (Active Area) or emerging trends. As of 2023, 62 documents still experience citation bursts (**Figure 9B** displays only the top 15), with 6 in #2 Cancer Vaccination, 25 in #3 Binding Domain, and 29 in #5 mRNA Vaccine, indicating these areas as key current and future focuses of nanovaccine research.

## 4 Discussion

### 4.1 General information

Over the past two decades, nanovaccine research has advanced through four distinct phases. Since 2020, studies in this field have rapidly progressed. An examination of keywords and references highlights its association with the COVID-19 pandemic. Numerous studies attest to the myriad advantages conferred by the application of nanotechnology in vaccine development, which is increasingly acknowledged as a primary strategy.

Researchers from 85 countries and regions have engaged in nanovaccine studies, highlighting its global prominence. The top 10 countries collectively authored 1923 articles, comprising 94.54% of all publications. The USA and China significantly outnumber other nations, with the USA spearheading due to substantial funding, close cooperation, and initiatives like the National Nanotechnology Initiative and Operation Warp Speed, bolstered by advanced research infrastructure and skilled researchers. China demonstrates rapid progress, supported by funding and an innovation-driven development strategy. International collaboration in nanovaccine research remains limited, with only 26.11% of articles being collaborative. The USA acts as a central hub for international cooperation, particularly with China, reflecting mutual research synergy and shared interests conducive to advancing the field. Thematic trend analysis reveals the focal points of countries in nanovaccine research. The USA has shown significant engagement in COVID-19 research, driven by its rapid scientific responsiveness and substantial resource allocation. China’s focus on areas like tumor immunotherapy reflects its investment in applied foundational research, possibly aligned with its long-term scientific strategy. The “13th Five-Year Plan “and” 14th Five-Year Plan” from the Chinese government plan to enhance research in life sciences and biotechnology, especially in cancer treatment and immunology. This diversity enriches global nanovaccine research, offering opportunities for future studies to explore specific domains more deeply by leveraging their unique strengths and requirements.

Universities play a pivotal role in this context. Among the top 10 institutions, a majority are situated in China and the USA. The Chinese Academy of Sciences stands out with 65 publications, highlighting its pivotal role in advancing research in this domain. However, in terms of citation counts, the University of California, San Diego holds greater sway, particularly in the research of nanovaccines for COVID-19 and cancer. The Howard Hughes Medical Institute and the Catholic University of Louvain consistently produce high-quality research, garnering widespread recognition. In contrast, institutions such as the Pasteur Institute of Iran and Tehran University Medical School, though more productive, show lower citation rates, potentially indicating reduced international impact. The emergence of prominent institutions in the nanovaccine domain correlates with their significant research prowess, increased financial backing, and enhanced collaborative efforts with other entities. Moreover, these findings highlight that in nanovaccine research, productivity does not always correlate with influence, a crucial consideration for identifying impactful institutions and potential collaborators. Collaborative networks among institutions demonstrate that geographical proximity facilitates inter-institutional cooperation, offering diverse advantages in nanovaccine research, including interdisciplinary synergy, resource sharing, innovation, and technology transfer.

Zhao Kai and Jin Zheng from Heilongjiang University are notably productive, focusing on research involving the Newcastle disease virus and chitosan nanoparticles. Wim Jiskoot from Leiden University commands the highest citation count, with research primarily concentrated on mRNA vaccines, chitosan nanospheres, PLGA, and nasal vaccination. Additionally, Veronique Preat, Marie Garinot, and Ligeng Xu have authored highly cited papers in this area. In scientific research, quality often outweighs quantity; studies with high citation rates typically demonstrate innovation and substantial practical value, advancing the field. Veronique Preat and Marie Garinot collaborate as a research duo dedicated to investigating oral vaccines, mRNA vaccines, and LNPs. Liu Ye holds the highest h-index, indicative of influential contributions to nanovaccine research, particularly HIV-1 vaccines. Co-citation networks and clustering analyses facilitate the identification of influential authors, offering critical insights and collaboration opportunities. Bachmann, MF, and Zhao L are influential in PLGA nanoparticle and mRNA nanovaccine research, respectively.

The analysis shows that the top ten journals rank at Q2 or higher in JCR, emphasizing the crucial role of prestigious and influential journals in advancing nanovaccine research. *Vaccine* has published the most articles and garnered the highest co-citations, demonstrating its broad recognition and impact in the field. *Journal of Controlled Release* reigns supreme in citation count and h-index, ranking second in publication output and co-citation. It boasts a distinguished impact factor and consistently disseminates research findings in the realm of nanovaccines. The journal has published an abundance of studies concerning adjuvants, chitosan, immunogenicity, and PLGA. Other prominent journals include *Biomaterials* and *ACS Nano.* Recent notable contributors include *Frontiers in Immunology* and *Vaccines*. Submitting relevant articles to these publications is a commendable choice. Moreover, focusing on current research themes in high-impact journals facilitates exploring new applications of nanomaterials and technologies. OverlayMaps illustrate citation relationships across diverse disciplines, emphasizing the interdisciplinary nature of nanovaccine research.

Financial support is crucial in nanovaccine research, with a majority of scholarly articles relying on diverse funding sources. Since 2014, funding for nanovaccine research has significantly increased, with over 80% of published papers in this field receiving financial backing. This trend underscores the growing importance of financial support over time and highlights the increasing financial demands and interdependencies within this research domain. The National Natural Science Foundation of China stands out as a principal funding source, facilitating significant achievements and sustained advancements in China’s contributions to this scientific field. Disparities in funding proportions among nations underscore the global unevenness and complexity of nanovaccine research. Future studies could further enhance international collaboration and financial integration to expedite the development and application of nanovaccine technologies.

### 4.2 Critical research topics in the domain of nanovaccines

The analysis of keywords and references yields valuable insights into the primary research focus. Dendritic cells and immune responses emerge as basic themes in nanovaccine research. Dendritic cells, as pivotal antigen-presenting cells within the immune system, uniquely capture, process, and present antigens, thereby activating and modulating specific T cell immune responses, particularly antigen-specific cytotoxic T lymphocytes (47). This capability renders dendritic cells ideal candidates for targeted vaccine strategies. Future research should explore optimizing and utilizing dendritic cell targeting systems to achieve more effective immunotherapies and vaccination strategies.

Analysis of high-frequency keywords highlights the primary nanocarriers used in nanovaccine research, encompassing a range of materials: Chitosan, virus-like particles, gold nanoparticles, microparticles, liposomes, microspheres, PLGA, and lipid nanoparticles. The diseases primarily under scrutiny comprise SARS-CoV-2, cancer (melanoma, breast cancer), influenza, and HIV. The top 3 clusters of the keywords (SARS-CoV-2, cancer immunotherapy, nanoparticles) delineate the three critical domains in nanovaccine research. #0 SARS-CoV-2 underscores urgent research imperatives prompted by the pandemic and underscores the pivotal role of nanotechnology in combating emerging viruses. #1 cancer immunotherapy emphasizes nanotechnology’s potential applications and significance in transformative treatment strategies. #2 nanoparticle highlights the fundamental and widespread applications of nanotechnology, crucially supporting vaccine and drug development.

Analyzing highly cited literature facilitates identifying research outcomes of significant impact and offers crucial insights for future research directions. The top 100 highly cited publications underscore the importance of foundational research and theoretical reviews in the field of nanovaccines. However, they also highlight that clinical research in nanovaccines is still in its early stages, suggesting considerable potential for advancement. Numerous highly cited articles investigate the physicochemical properties of vaccine delivery vectors and nanoparticles, emphasizing their fundamental role in nanovaccine research. Other influential publications also address nanovaccine development for diseases such as cancer, COVID-19, hepatitis B, influenza, and malaria, highlighting the wide-ranging application of nanovaccines in disease prevention and treatment.

The analysis of keywords and co-citation clusters reveals evolving focal points and developmental trends in nanovaccine research. Initial discourse on nanovaccines predominantly centered on nasal vaccinations. Early contributions from Jiskoot Wim have notably enriched this domain. Nevertheless, the formulation of nasal vaccines presents challenges, such as the existence of nasal barriers and the associated risks of brain entry (18). Subsequently, subunit vaccine antigens gained greater traction by utilizing specific pathogen portions (subunits) as antigens, minimizing adverse reactions and enhancing control over vaccine production (48). Integrating nanotechnology and adjuvants has propelled the development of subunit antigen vaccines, proving pivotal in recent pandemics (49, 50). However, the 2019 onset of the COVID-19 pandemic prompted a substantial reallocation of research resources towards COVID-19 vaccine development (51). This shift swiftly brought LNPs and mRNA technologies into sharp focus, introducing a new research direction across various nanovaccine domains (52). Furthermore, the rise of cancer immunotherapy has spurred research in cancer nanovaccines, culminating in a zenith of studies around 2017, a trend that persists. Recent studies have been influenced by precision medicine, with personalized cancer nanovaccines emerging as a burgeoning trend. These transitions underscore the field’s dynamic adaptation to emerging scientific insights and accentuate the imperative of recalibrating treatment paradigms in response to these advancements.

### 4.3 Hotspots and frontiers of nanovaccine

The aforementioned analysis indicates that “cancer nanovaccines,” “LNPs,” “mRNA nanovaccines,” and “COVID-19 nanovaccines” are pivotal areas of current and future research. Key research focuses include “candidate vaccines,” “protein nanoparticle,” “cationic lipids,” “ionizable lipids,” “machine learning,” “long-term storage,” “personalized cancer vaccines,” “neoantigens,” “outer membrane vesicles,” “*in situ* nanovaccine,” and “biomimetic nanotechnologies.”

The analysis of the #3 binding domain and #5 mRNA vaccines elucidates the developmental trajectory of COVID-19 nanovaccines, underscoring the pivotal role of LNPs in mRNA delivery. The COVID-19 pandemic has spurred extensive clinical trials for COVID-19 vaccines (53), with the USA and China leading this field. The advantages of LNPs, including safety and rapid development, combined with the adaptability of mRNA vaccines, are well-suited for actively mutating pathogens such as SARS-CoV-2 (15, 16), thus culminating in the success attained by the Pfizer/BioNTech and Moderna COVID-19 vaccines (7, 8). This sparked significant interest in nanotechnology for the development of novel vaccines. LNPs play a pivotal role in the preparation of COVID-19 mRNA vaccines, contributing to mRNA protection, intracellular escape, and targeted delivery (54). Presently, amidst the enduring global COVID-19 crisis and apprehensions regarding vaccine efficacy against emergent variants (55), attention remains fixated on “candidate vaccines,” with LNP-mRNA vaccines maintaining their preference. Some research endeavors concentrate on optimizing LNPs to enhance the efficacy of mRNA vaccines. This necessitates a thorough investigation into the structural-functional interplay among the lipid components of LNPs. The “ionizable lipid” has emerged as a research hotspot (56, 57), a crucial component of LNPs, exerting significant influence on processes such as endocytosis and cellular uptake, thereby affecting adjuvant effects and immune responses (58). The integration of machine learning expedites LNP optimization (59). Additionally, the storage prerequisites of mRNA vaccines (ultra-cold supply chains) present a logistical challenge in global distribution (60), hence inciting a fervent interest in developing more convenient storage modalities (61). Substantial progress has been achieved in the field of “lyophilization. (61, 62)In the future, in addition to optimizing the formulation and stability of LNPs, it is crucial to investigate the long-term safety and *in vivo* mechanisms of LNP-mRNA vaccines, such as the distribution and metabolic pathways of LNPs in various tissues and cell types, to enhance their design and application. Furthermore, the analysis of burst keywords reveals that “protein nanoparticle” has also recently become a focal point. It has emerged as a promising platform for presenting the S protein antigen (63, 64), with ferritin being the most commonly used non-viral self-assembling protein in research and clinical settings. Ferritin offers numerous advantages, including facilitating multivalent vaccine formulation, high stability, eliminating cold chain transportation, ease of production, and low cost, indicating its immense potential for future application in COVID-19 nanovaccines (65, 66).

In 2011, the FDA’s approval of Ipilimumab for melanoma treatment marked a pivotal moment in cancer immunotherapy (67), fueling increased research focus on cancer nanovaccine. Reputable journals, including *Biomaterials* and *ACS Nano*, have extensively covered these advancements. Analyzing the core reference of #2 cancer immunotherapy reveals several key directions and breakthroughs in the field of cancer nanovaccines, such as the enhancement of targeted delivery through the “albumin hitchhiking” strategy (28), “*in situ* vaccination” strategy (68, 69), biomimetic nanotechnologies involving cancer cell membranes (32, 70), erythrocyte membrane nanovesicles (71, 72), and novel immunomodulatory pathways (STING activation) (30, 73, 74).Recently, advancements in high-throughput sequencing and epitope prediction algorithms have thrust personalized cancer vaccines into the spotlight (75, 76). Tumor neoantigens exhibit tumor-specific and highly immunogenic properties, offering novel methods for precise targeting and elimination of cancer cells (77). Additionally, the COVID-19 pandemic has prompted a reassessment of cancer vaccine research, with various LNP-mRNA-based cancer vaccines currently undergoing clinical trials (78). In the future, the continuous development of nanovaccines will unfold new possibilities for personalized oncology (79). Nevertheless, future investigations into the potential toxicity and *in vivo* mechanisms of nanovaccines are crucial, necessitating substantial efforts to translate cancer vaccines from the laboratory to clinical application.

### 4.4 Strengths and limitations

This study represents the inaugural application of bibliometrics to scrutinize and delineate the historical knowledge landscape surrounding ‘nanovaccines,’ offering a visual portrayal of research distribution, focal points, and trends. However, it is imperative to acknowledge several limitations: 1) Solely incorporating WOSCC data could potentially introduce selection bias. 2) Bibliometric methodologies, reliant on natural language processing, may entail errors. Nevertheless, this research remains valuable for readers interested in understanding the current status, focal points, and trends in the nanovaccine field. Future studies will broaden the scope of literature analysis by incorporating data from additional journal databases such as Scopus.

## 5 Conclusion

The field of nanovaccines is undergoing rapid expansion and is poised to remain a significant focal point in the foreseeable future. This study employs bibliometric analysis to investigate nanovaccine literature spanning the past four decades, yielding the following findings:

1. The USA and China have made substantial contributions to this research domain and maintain robust collaboration, with the USA serving as a pivotal center for international cooperation. The primary contributors are predominantly academic institutions, with notable prominence from institutions in both the USA and China. Noteworthy mentions include the Chinese Academy of Sciences, which leads in publication frequency, and the University of California, San Diego, which ranks highest in citation impact. Other key institutions include the Howard Hughes Medical Institute and the Catholic University of Louvain. Among scholars, Zhao Kai from China has authored the most articles, while Wim Jiskoot from Leiden University garners the highest citation count. Additional notable authors include Veronique Preat, Marie Garinot, Ligeng Xu, and Liu Ye. “*Vaccine*” claims the highest publication volume, while “*Journal of Controlled Release*” holds the greatest influence. Contributions also emanate from journals such as “*Frontiers in Immunology*” and “*Vaccines*”. Funding support significantly propels advancements in this research field.
2. Co-occurrence analysis of keywords reveals that prominent nanocarriers used in nanovaccine research include chitosan, virus-like particles, gold nanoparticles, liposomes, PLGA, and lipid nanoparticles. Nanovaccines are primarily designed to combat diseases such as SARS-CoV-2, various cancers, influenza, and HIV. Dendritic cells and immune responses are foundational topics within nanovaccine research.
3. Analysis of highly cited literature delves into the physicochemical properties of vaccine delivery vehicles and nanoparticles, alongside studies on tumors, COVID-19 vaccines, hepatitis B, influenza, malaria, and other conditions. It emphasizes the critical role of fundamental research and theoretical reviews in the field of nanovaccines, indicating that clinical studies in this area are still in an early developmental phase with significant growth potential.
4. Clustering analysis of co-citation networks identifies nine primary clusters, vividly illustrating the evolution of research themes over different periods. Highly co-cited articles underscore the profound impact of mRNA vaccines on nanovaccine research. Citation bursts indicate that cancer vaccines, binding domains, and mRNA vaccines are pivotal areas of focus for current and future research in nanovaccines.

In summary, the bibliometric analysis of nanovaccine literature delineates the evolutionary trajectory of cumulative knowledge over the past forty years and emphasizes future research avenues aimed at proactive approaches to vaccine design and addressing challenges posed by infectious diseases. Additionally, using CiteSpace, VOSviewer, and R-bibliometrix underscores the potential for reproducibility and validation with new data, based on selected computational attributes.

## Data Availability

The original contributions presented in the study are included in the article/**Supplementary Material**. Further inquiries can be directed to the corresponding authors.
