# Supplementary material for: A bibliometric insight into nanomaterials in vaccine: trends, collaborations, and future avenues
Source: Front Immunol. 2024 Aug 12;15:1420216. doi: 10.3389/fimmu.2024.1420216 (PMC11345159; doi:10.3389/fimmu.2024.1420216)
Supplement: Supplementary file 1 [file DataSheet1.zip › supplementary material 1/The detailed information of Bibliometric Analysis.docx]

# The detailed information of “3.1.2 Bibliometric Analysis and Visualization”

1.The overview of Bibliometric Analysis and Visualization

| **Tool** | **Function** | **Default Parameter Configuration** | **Adjustment of other Parameters** | |
| --- | --- | --- | --- | --- |
| Microsoft Excel 2019 | line graphs (Figure 1A);  bar graphs (Figure 2C)(Figure 6);  all tables | | | |
| Citespace | overlayMaps of journals  (Figure 5D) | | Enable the overlay function for journals in the “JCR Journal Maps” under the “OverlayMaps” menu, and merge connections using the “Z-score” functionality. | |
|  | Keywords:  burst analysis(Figure 7C);  cluster analysis(Figure 7D) | Timespan=1981–2023;  Years per slice=1;  Links:Strength(Cosine), Scope(Within Slices);  Selection criteria:g-index (k=25), Pruning :no;  Show cluster labels:log-likelihood ratio(LLR) | 1. Node type = keywords;  2.Each keyword cluster was named using keywords with the highest log-likelihood ratio. | |
|  | co-cited references:  cluster analysis(Figure 8A, Figure 8B),  time zone graph(Figure 8C),  burst analysis(Figure 9) |  | 1. Node type = reference;  2. Due to the absence of keywords in Web of Science (WOS) prior to 1990, terms were extracted from titles, and each co-citation cluster was named using title words with the highest log-likelihood ratio.  3.Display the largest K clusters:9 | |
|  | co-cited authors(Table 2) |  | 1. Node type = cited authors;  2. Due to the absence of keywords in Web of Science (WOS) prior to 1990, terms were extracted from titles, and each co-citation cluster was named using title words with the highest log-likelihood ratio. | |
| VOSviewer | Co-authorship network:  countries(Figure 2A),  institutions(Figure 3A) | Counting method: Full counting; | VOSviewer thesaurus file (optional): “thesaurus_country” | |
|  | Co-authorship network:  institution (Figure 3B),  author(Figure 4A); |  | No | |
|  | Co-citation:  cited author (Figure 4B),  cited source(Figure 5C) |  | No | |
|  | Co-occurrence of keywords  (Figure 7A) |  | Min. cluster size:35 | |
|  | Co-occurrence of journal-keyword  (Figure 5B) |  | Choose threshold(Minimum number of occurrences of a keyword/journal:10) | |
|  | Co-occurrence of terms  (Figure 1B, Figure 2E, Figure 2F, Figure 3D, Figure 3E) | Fields from which terms will be extracted:Title and abstract;  Ignore structured abstract labels ; Ignore copyright statements ;  Counting method: Binary counting | No | |
| Pajek | Co-occurrence of keywords  (Figure 7A) | Firstly, export the keyword co-occurrence data from VOSviewer into a Pajek format file.  Secondly, utilize Pajek's Network + FirstPartition + First Vector functions, employing the "In y Direction" layout to vertically arrange keyword nodes based on clustering.  Thirdly, export the results back into VOSviewer. | | |
| Scimago Graphica | Geographic visualization of co-authorship network:  countries  (Figure 2A) | Firstly, export the GML format file of co-authorship network from VOSviewer;  Secondly, configure the parameters:  Size: document;  Color: citations; | Label:country, document, citations;  Marks:disks(layout:map); | |
|  | co-authorship network:  institutions(Figure 3A) |  | Label:institution, document, citations;  Marks:disks(layout:Circular) | |
| R-bibliometrix | H_index and M_index:journal, author  (Table 1, Table 3);  Number of documents from corresponding authors' countries(Figure 2B);  Thematic maps (Figure 7E, 7F) | Importing data into R-bibliometrix yields results directly. | | |
|  | H_index and M_index:country | 1. Import the plain text data exported from WOS into R-Bibliometrix, and export it as a CSV file named “Bibliometrix-Export-File-2023-11-09”.  2. Use R-studio to read the “Bibliometrix-Export-File-2023-11-09.csv” and write the corresponding code to compute the results. | | code |
|  | Yearly occurrences:top 30 countries, institutions, journals, keywords (Figure 2D;3C;5A;7B); |  |  |  |
|  | Proportion trends for the top 10 countries' publications(Figure 2C). |  |  |  |

**2.**

Moreover, the implementation of two functionality is intricate, requiring a flexible understanding of the structure of the WoS dataset and the principles of VOSviewer.

**2.1 Network Overlay Analysis**

VOSviewer enables the superimposition of specific data subsets onto the overall dataset, such as constructing a network of term co-occurrences for a given period across the entire network. In this study, Figures 1B, 2E, 2F, 3D, and 3E utilized this functionality. Taking Figure 1 as an illustrative example:

Firstly, during the initial data collection phase, besides gathering all literature comprehensively, documents were also collected annually.

Secondly, employing VOSviewer to construct the overall term co-occurrence network of nanovaccine research, followed by clicking "save" to preserve the map file and network file from VOSviewer, saved as map1.csv and net1.txt.

Thirdly, utilizing VOSviewer to import data from 2020 to 2023 and generate the term co-occurrence network for these years. Subsequently, clicking "save" saves the two files map2.csv and net2.txt.

Fourthly, opening map1.csv in Excel, where, due to this study's utilization of color variation to denote proportional sizes, the 'score' field in the map file represents color. Thus, creating a new column "score<term Occurrences>" and utilizing Excel's "VLOOKUP" function to match the term frequencies from map2.csv to the "score<term Occurrences>" column in map1.csv.

Fifthly, creating a new column "score<term scale>" in map1.csv, dividing the frequencies in "score<term Occurrences>" by the total term occurrences in the 'weight<Occurrences>' column, obtaining proportions, and filling in the "score<term scale>" column. Ultimately, this yields the contribution proportions of nanovaccine research terms from 2020 to 2023 relative to the overall nanovaccine research.

Sixthly, saving the modified map1.csv as a "Text (tab delimited)(*.txt)" file, named map1.txt.

Seventhly, using VOSviewer's "open" function to import map1.txt and net1.txt, switching to the "Overlay Visualization" interface, selecting the 'scores' function in the Visualization Operations interface, and choosing the newly added "score<term scale>" for colors, selecting "Rainbow" for the colors option, thereby obtaining a visual representation of the contribution proportions of nanovaccine research terms from 2020 to 2023 relative to the overall nanovaccine research.

Other overlays based on VOSviewer operate similarly.

**2.2 Co-occurrence of journal-keyword**

The co-occurrence network of journal-keyword is dependent on plain text data exported from WoS. This data comprises 68 fields, including SO (source), DE (author keywords), and ID (keywords Plus), which are crucial for constructing the co-occurrence network of journal-keyword.

Firstly, exchange the SO and ID fields.

Secondly, utilize VOSviewer's “co-occurrence” function.

Note: It should be clarified that the keywords in this co-occurrence network of journal-keyword consist solely of author keywords, distinct from the content of keywords in Co-occurrence of keywords(Figure 7A). VOSviewer's “co-occurrence” function is unique in its ability to identify co-occurrences between two fields,.

**2.3** Code of Yearly occurrences figure

**2.3.1** Yearly occurrences of the top 30 keywords

setwd("D:\\桌面\\纳米疫苗\\R分析\\总文件")

library(reshape2)

library(tidyverse)

library(bibliometrix)

#数据读入法

Bw<-read.csv("Bibliometrix-Export-File-2023-11-09.CSV")#biblioshiny获取

#同义词合并

synonyms <- c("NANOPARTICLES; NANOPARTICLE",

"VACCINE ADJUVANT; ADJUVANT; ADJUVANTS",

"vaccine; vaccines",

"SARS-COV-2; COVID-19",

"NANOVACCINE; NANOPARTICLE VACCINE",

"CHITOSAN; CHITOSAN NANOPARTICLES; CHITOSAN NANOPARTICLE",

"VIRUS-LIKE PARTICLES; VLPS; VLP",

"PLGA; PLGA NANOPARTICLES; PLGA-BASED NANOPARTICLES; POLY(LACTIC-CO-GLYCOLIC ACID); PLGA NANOPARTICLE",

"LIPOSOMES; LIPOSOME",

"LIPID NANOPARTICLES; LIPID NANOPARTICLE")

topKW=KeywordGrowth(Bw, Tag = "DE", sep = ";", top=30, cdf=FALSE,synonyms=synonyms)

topKW2=melt(topKW, id='Year')

#折线图

ggplot(topKW2,aes(Year,value, group=variable, color=variable))+geom_line()+theme_bw()

#标准化

library(plyr)

library(scales)

data <- ddply(topKW2, .(variable),transform,rescale = rescale(value))

#rescale()函数内默认参数to = c(0,1)，所以标准化范围也可以自定义

#热图

library("viridis")#渐变色

ggplot(data, aes(Year, variable)) +

geom_tile(aes(fill =rescale),colour = "white") +

#scale_fill_distiller(palette="Spectral",name="value") +

#scale_fill_gradientn(values = seq(0,1,0.2),colours = c('cyan','blue','green','orange','red'),name = "value")+

#scale_fill_viridis(option = "A",name = "value")+

scale_fill_viridis(option = "B",name = "value")+

#scale_fill_viridis_c(name = "value")+

coord_fixed(ratio=1)+

scale_x_continuous(expand = c(0,0))+#这个可以去掉与X轴间隙

scale_x_continuous(expand=c(0,0),

breaks=seq(1981, 2023, by=1)) +

theme_bw()+

theme(axis.text.x = element_text(angle=90,vjust = 0.5, hjust = 0.5))+

theme(axis.text= element_text(size = 13,family="serif"))+

theme(axis.title=element_text(size=14,face="bold"))+

labs(x = "Year", y = "Author Keywords", title = "Keywords Distribution Over Time")+

theme(plot.title = element_text(size = 15,hjust = 0.5,face="bold"))+

theme(legend.key.width=unit(3,'mm'),legend.key.height=unit(3,'cm'))+

theme(legend.title = element_text(size = 10,family="serif"))+

theme(panel.grid.major =element_blank(),

panel.grid.minor = element_blank(),

panel.background = element_blank(),#去除背景

panel.border = element_blank())#去除边框

## Yearly occurrences of the top 30 countries

setwd("D:\\桌面\\纳米疫苗\\R分析\\总文件")

**library**(reshape2)

**library**(tidyverse)

**library**(bibliometrix)

#数据读入法二

M<-read.csv("Bibliometrix-Export-File-2023-11-09.CSV")#biblioshiny获取

A<-biblioAnalysis(M)

sep=";"

**if** (!("AU_CO" %**in**% names(M))){

M=metaTagExtraction(M,Field="AU_CO",sep)}

topCO<-KeywordGrowth(

M,

Tag = "AU_CO",

sep = ";",

top = 30,

cdf = F,

remove.terms = NULL,

synonyms = NULL

)

topCO2=melt(topCO, id='Year')

#折线图

ggplot(topCO2,aes(Year,value, group=variable, color=variable))+geom_line()+theme_bw()

#标准化

**library**(plyr)

**library**(scales)

data <- ddply(topCO2, .(variable),transform,rescale = rescale(value))

#rescale()函数内默认参数to = c(0,1)，所以标准化范围也可以自定义

#热图

**library**("viridis")#渐变色

ggplot(data, aes(Year, variable)) +

geom_tile(aes(fill =rescale),colour = "white") +

#scale_fill_distiller(palette="Spectral",name="value") +

#scale_fill_gradientn(values = seq(0,1,0.2),colours = c('cyan','blue','green','orange','red'),name = "value")+

#scale_fill_viridis(option = "A",name = "value")+

scale_fill_viridis(option = "B",name = "value")+

#scale_fill_viridis_c(name = "value")+

coord_fixed(ratio=1)+

scale_x_continuous(expand = c(0,0))+#这个可以去掉与X轴间隙

scale_x_continuous(expand=c(0,0),

breaks=seq(1981, 2023, by=1)) +

theme_bw()+

theme(axis.text.x = element_text(angle=90,vjust = 0.5, hjust = 0.5))+

theme(axis.text= element_text(size = 13,family="serif"))+

theme(axis.title=element_text(size=14,face="bold"))+

labs(x = "Year", y = "Country", title = "Country Distribution Over Time")+

theme(plot.title = element_text(size = 15,hjust = 0.5,face="bold"))+

theme(legend.key.width=unit(3,'mm'),legend.key.height=unit(3,'cm'))+

theme(legend.title = element_text(size = 10,family="serif"))+

theme(panel.grid.major =element_blank(),

panel.grid.minor = element_blank(),

panel.background = element_blank(),#去除背景

panel.border = element_blank())#去除边框

## Yearly occurrences of the top 30 institutions

setwd("D:\\桌面\\纳米疫苗\\R分析\\总文件")

library(reshape2)

library(tidyverse)

library(bibliometrix)

#数据读入法

Bw<-read.csv("Bibliometrix-Export-File-2023-11-09.CSV")#biblioshiny获取

#同义词合并

synonyms <- c("UNIVERSITY OF WASHINGTON; UNIVERSITY OF WASHINGTON SEATTLE","UNIVERSITY OF TEXAS SYSTEM; UNIVERSITY OF TEXAS MEDICAL BRANCH GALVESTON","UNIVERSITY OF CALIFORNIA SYSTEM;UNIVERSITY OF CALIFORNIA SAN DIEGO")

topKW=KeywordGrowth(Bw, Tag = "AU_UN", sep = ";", top=30, cdf=FALSE,synonyms=synonyms)

topKW2=melt(topKW, id='Year')

#折线图

ggplot(topKW2,aes(Year,value, group=variable, color=variable))+geom_line()+theme_bw()

#标准化

library(plyr)

library(scales)

data <- ddply(topKW2, .(variable),transform,rescale = rescale(value))

#rescale()函数内默认参数to = c(0,1)，所以标准化范围也可以自定义

#热图

library("viridis")#渐变色

ggplot(data, aes(Year, variable)) +

geom_tile(aes(fill =rescale),colour = "white") +

#scale_fill_distiller(palette="Spectral",name="value") +

#scale_fill_gradientn(values = seq(0,1,0.2),colours = c('cyan','blue','green','orange','red'),name = "value")+

#scale_fill_viridis(option = "A",name = "value")+

scale_fill_viridis(option = "B",name = "value")+

#scale_fill_viridis_c(name = "value")+

coord_fixed(ratio=1)+

scale_x_continuous(expand = c(0,0))+#这个可以去掉与X轴间隙

scale_x_continuous(expand=c(0,0),

breaks=seq(1981, 2023, by=1)) +

theme_bw()+

theme(axis.text.x = element_text(angle=90,vjust = 0.5, hjust = 0.5))+

theme(axis.text= element_text(size = 6,family="serif"))+

theme(axis.title=element_text(size=14,face="bold"))+

labs(x = "Year", y = "Institution", title = "Institution Distribution Over Time")+

theme(plot.title = element_text(size = 15,hjust = 0.5,face="bold"))+

theme(legend.key.width=unit(2,'mm'),legend.key.height=unit(1.5,'cm'))+

theme(legend.title = element_text(size = 10,family="serif"))+

theme(panel.grid.major =element_blank(),

panel.grid.minor = element_blank(),

panel.background = element_blank(),#去除背景

panel.border = element_blank())#去除边框

## Yearly occurrences of the top 30 journals

setwd("D:\\桌面\\纳米疫苗\\R分析\\总文件")

library(reshape2)

library(tidyverse)

library(bibliometrix)

#数据读入法

Bw<-read.csv("Bibliometrix-Export-File-2023-11-09.CSV")#biblioshiny获取

topKW=KeywordGrowth(Bw, Tag = "SO", sep = ";", top=30, cdf=FALSE)

topKW2=melt(topKW, id='Year')

#折线图

ggplot(topKW2,aes(Year,value, group=variable, color=variable))+geom_line()+theme_bw()

#标准化

library(plyr)

library(scales)

data <- ddply(topKW2, .(variable),transform,rescale = rescale(value))

#rescale()函数内默认参数to = c(0,1)，所以标准化范围也可以自定义

#热图

library("viridis")#渐变色

ggplot(data, aes(Year, variable)) +

geom_tile(aes(fill =rescale),colour = "white") +

#scale_fill_distiller(palette="Spectral",name="value") +

#scale_fill_gradientn(values = seq(0,1,0.2),colours = c('cyan','blue','green','orange','red'),name = "value")+

#scale_fill_viridis(option = "A",name = "value")+

scale_fill_viridis(option = "B",name = "value")+

#scale_fill_viridis_c(name = "value")+

coord_fixed(ratio=1)+

scale_x_continuous(expand = c(0,0))+#这个可以去掉与X轴间隙

scale_x_continuous(expand=c(0,0),

breaks=seq(1981, 2023, by=1)) +

theme_bw()+

theme(axis.text.x = element_text(angle=90,vjust = 0.5, hjust = 0.5))+

theme(axis.text= element_text(size = 10,family="serif"))+

theme(axis.title=element_text(size=14,face="bold"))+

labs(x = "Year", y = "Journals", title = "Journals Distribution Over Time")+

theme(plot.title = element_text(size = 15,hjust = 0.5,face="bold"))+

theme(legend.key.width=unit(3,'mm'),legend.key.height=unit(3,'cm'))+

theme(legend.title = element_text(size = 10,family="serif"))+

theme(panel.grid.major =element_blank(),

panel.grid.minor = element_blank(),

panel.background = element_blank(),#去除背景

panel.border = element_blank())#去除边框
