# Supplementary figures and images for: A bibliometric insight into nanomaterials in vaccine: trends, collaborations, and future avenues
Source: Front Immunol. 2024 Aug 12;15:1420216. doi: 10.3389/fimmu.2024.1420216 (PMC11345159; doi:10.3389/fimmu.2024.1420216)

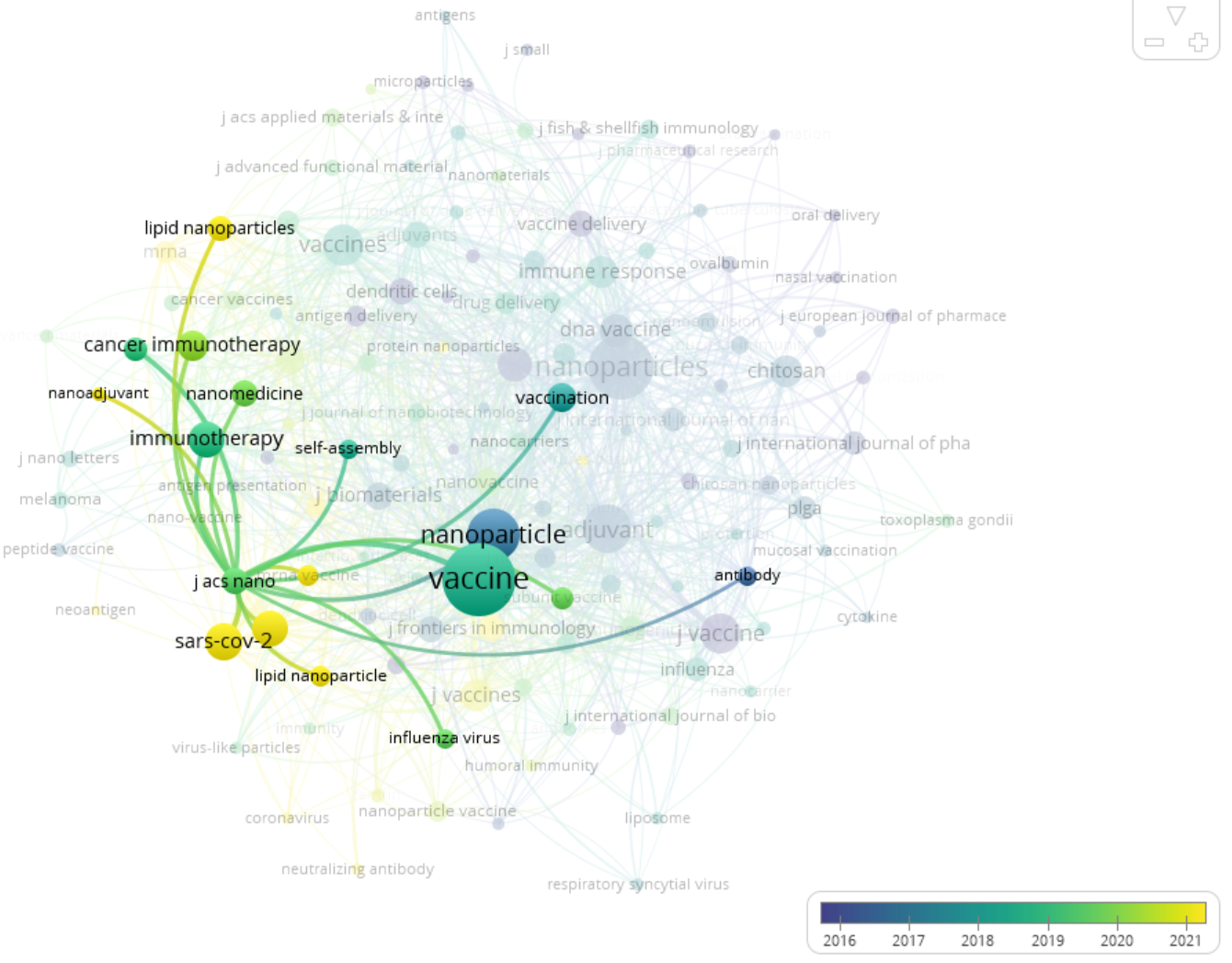

Supplement: Supplementary file 3 [file DataSheet3.zip › supplementary material 6/ACS Nano-keywords.png]

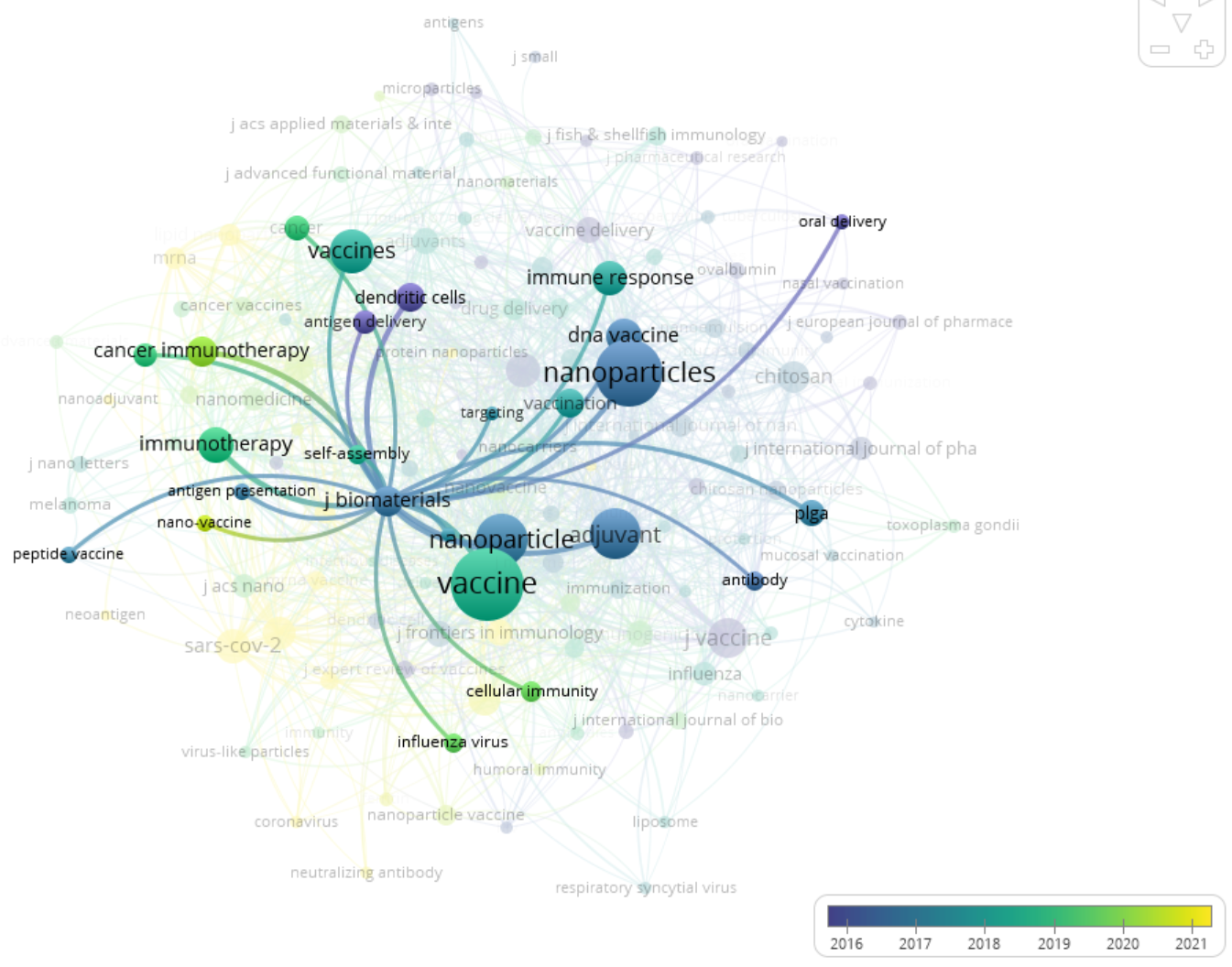

Supplement: Supplementary file 3 [file DataSheet3.zip › supplementary material 6/Biomaterials-keywords.png]

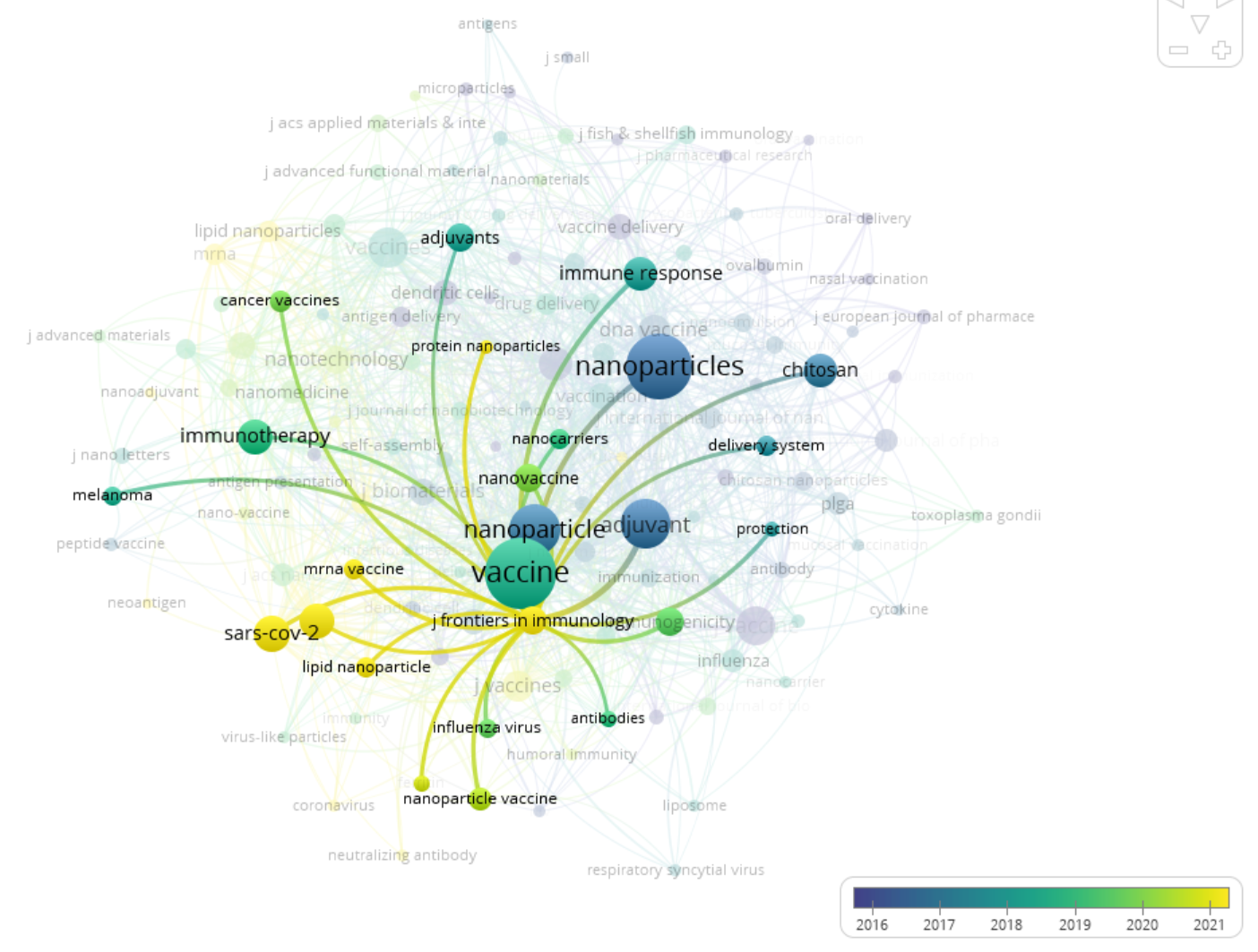

Supplement: Supplementary file 3 [file DataSheet3.zip › supplementary material 6/Frontiers in Immunology-keywords.png]

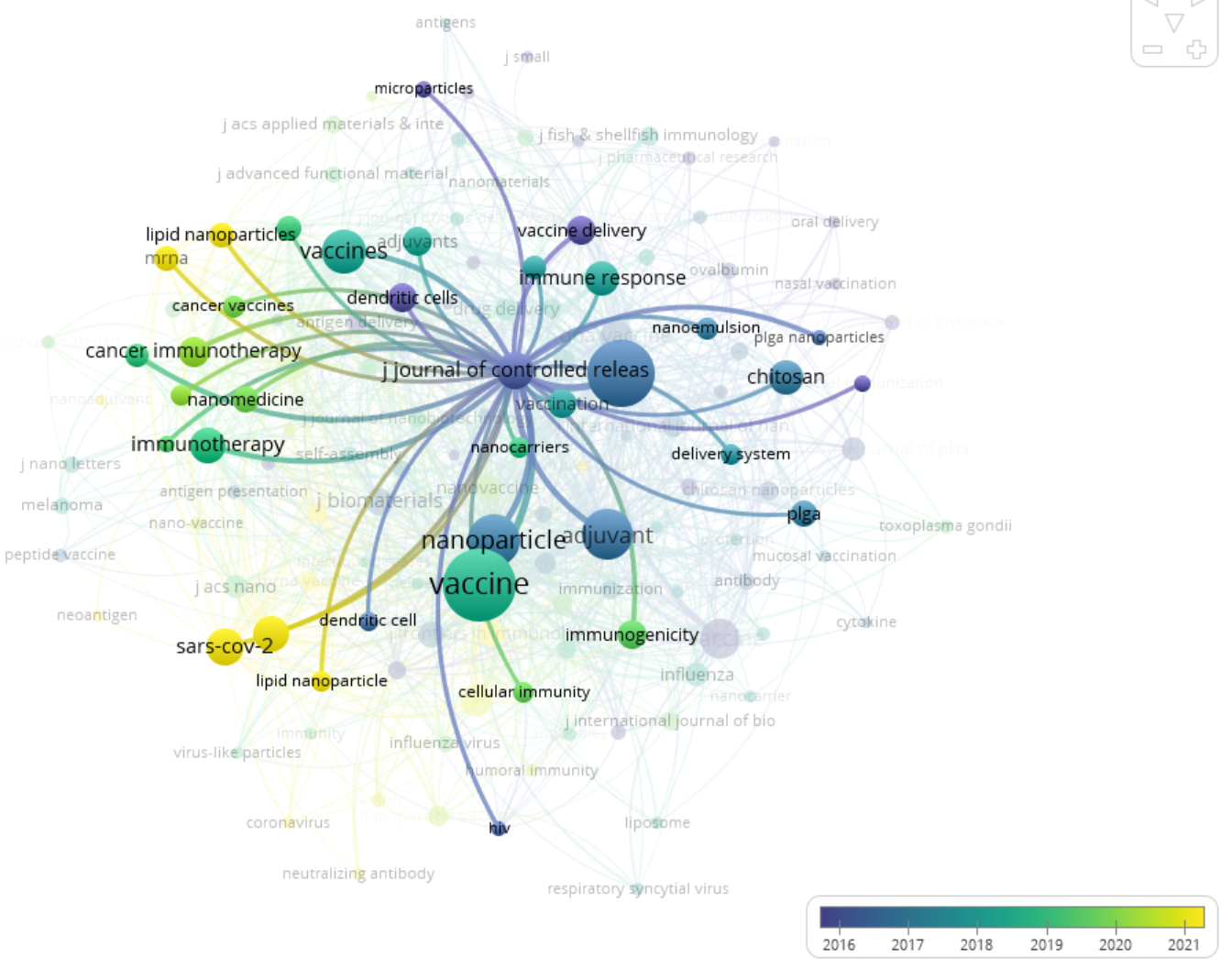

Supplement: Supplementary file 3 [file DataSheet3.zip › supplementary material 6/Journal of Controlled Release-keywords.png]

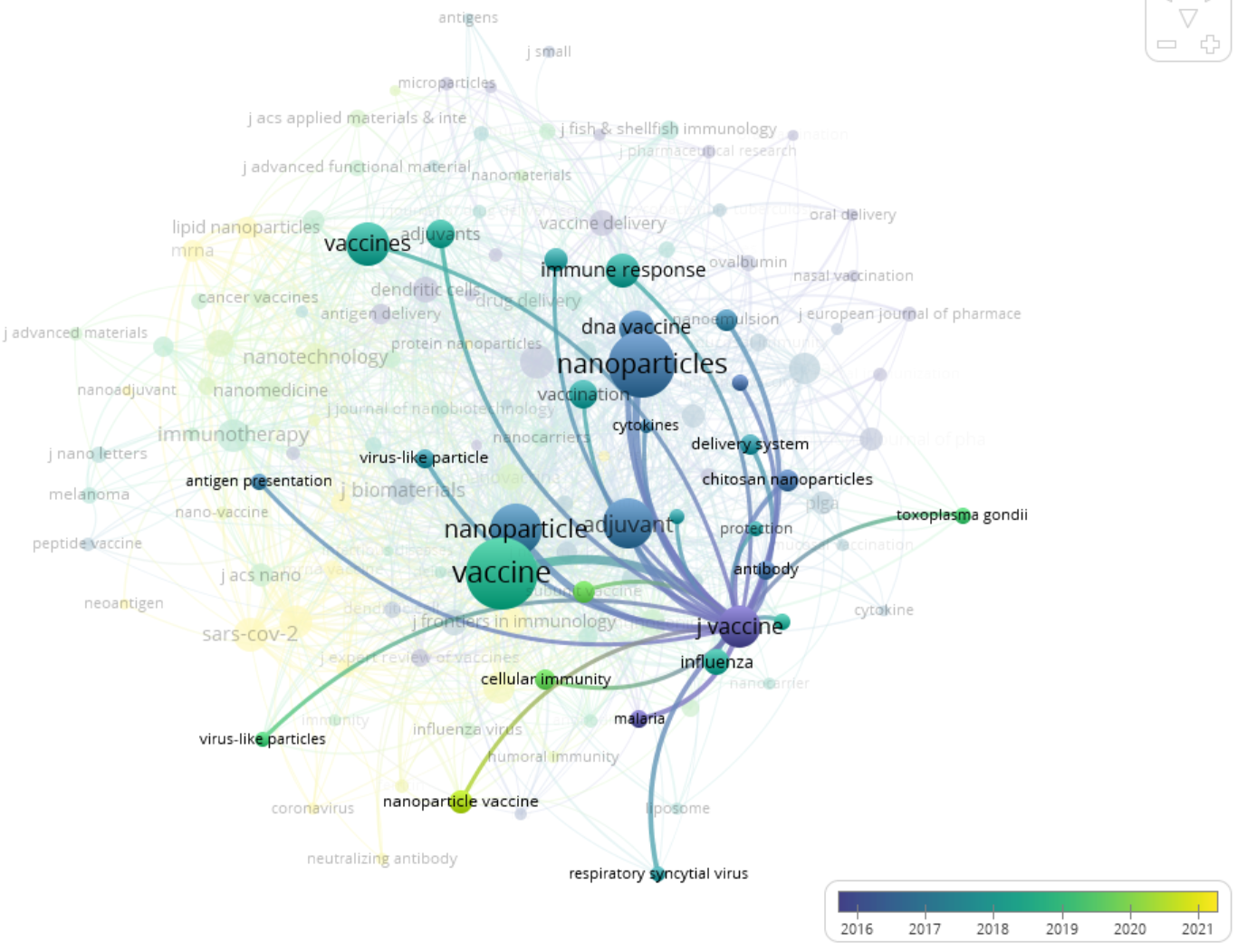

Supplement: Supplementary file 3 [file DataSheet3.zip › supplementary material 6/Vaccine-keywords.png]

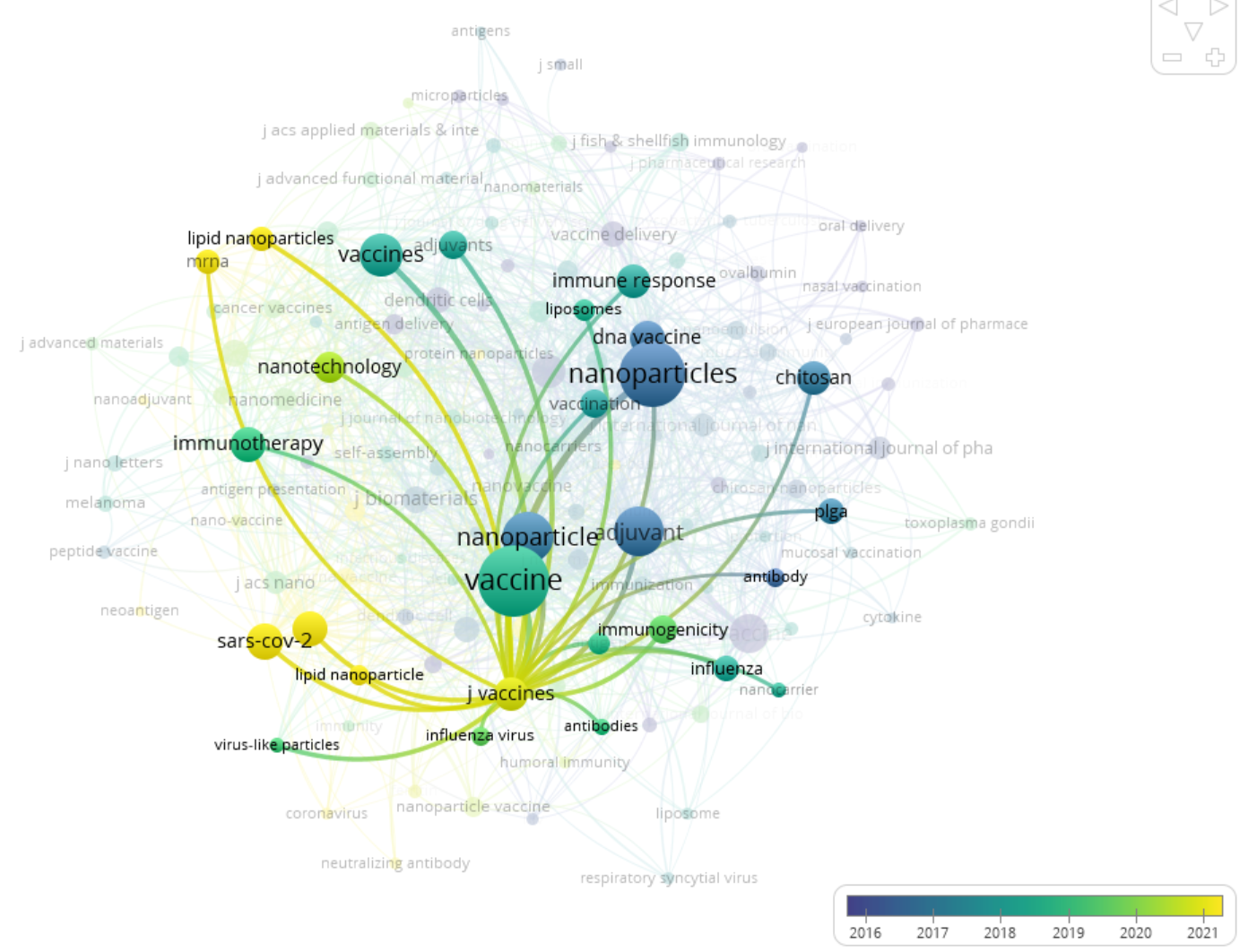

Supplement: Supplementary file 3 [file DataSheet3.zip › supplementary material 6/Vaccines-keywords.png]
